# Quantitative dissection of *Agrobacterium* T-DNA expression in single plant cells reveals density-dependent synergy and antagonism

**DOI:** 10.1038/s41477-025-01996-w

**Published:** 2025-05-12

**Authors:** Simon Alamos, Matthew J. Szarzanowicz, Mitchell G. Thompson, Danielle M. Stevens, Liam D. Kirkpatrick, Amanda Dee, Hamreet Pannu, Ruoming Cui, Shuying Liu, Monikaben Nimavat, Ksenia Krasileva, Edward E. K. Baidoo, Patrick M. Shih

**Affiliations:** 1 Joint BioEnergy Institute, Emeryville, CA USA; 2 Environmental Genomics and Systems Biology Division, Lawrence Berkeley National Laboratory, Berkeley, CA USA; 3 Department of Plant and Microbial Biology, University of California, Berkeley, CA USA; 4 Center for Computational Biology, University of California, Berkeley, CA USA; 5 Innovative Genomics Institute, Berkeley, CA USA

**Keywords:** Plant sciences, Genetics, Plant biotechnology, Microbiology

## Abstract

*Agrobacterium* pathogenesis, which involves transferring T-DNA into plant cells, is the cornerstone of plant genetic engineering. As the applications that rely on *Agrobacterium* increase in sophistication, it becomes critical to achieve a quantitative and predictive understanding of T-DNA expression at the level of single plant cells. Here we examine if a classic Poisson model of interactions between pathogens and host cells holds true for *Agrobacterium* infecting *Nicotiana benthamiana*. Systematically challenging this model revealed antagonistic and synergistic density-dependent interactions between bacteria that do not require quorum sensing. Using various approaches, we studied the molecular basis of these interactions. To overcome the engineering constraints imposed by antagonism, we created a dual binary vector system termed ‘BiBi’, which can improve the efficiency of a reconstituted complex metabolic pathway in a predictive fashion. Our findings illustrate how combining theoretical models with quantitative experiments can reveal new principles of bacterial pathogenesis, impacting both fundamental and applied plant biology.

## Abstract (web summary)

Alamos et al. show that *Agrobacterium* engages in density-dependent interactions that dictate transformation efficiency and transgene expression. These findings enable a quantitative model to improve metabolic engineering in a predictive manner.

## Main

Harnessing a unique aspect of the pathogenesis of *Agrobacterium*, the transfer of T-DNA into the plant cell nucleus, ushered in the era of plant biotechnology^1–3^. A particularly powerful and popular method to transiently express T-DNAs in plant cells consists of infiltrating bacterial cultures into *Nicotiana benthamiana* (tobacco) leaves, also known as agroinfiltration^4^. By mixing multiple *Agrobacterium* strains carrying different T-DNA constructs, it is possible to use agroinfiltration to quickly reconstitute complex metabolic pathways and multilayered synthetic gene circuits in plants^5–8^. *Agrobacterium* cultures carrying massive gene libraries have been used to characterize plant DNA regulatory regions in parallel using next-generation sequencing^9^. These are but a few examples of a growing repertoire of experiments of increasing sophistication that rely on *Agrobacterium* T-DNA transfer. Interpreting and optimizing these experiments depends on a quantitative and predictive understanding of *Agrobacterium* pathogenesis, particularly the number of strains that transform each host cell. Yet, despite the key role of this host–pathogen interaction for basic and applied plant biology, our grasp of this process remains largely qualitative. We lack a theoretical framework to model T-DNA expression in single plant cells.

At a more basic level, a quantitative dissection of *Agrobacterium* DNA transfer and expression will allow testing mechanistic hypotheses about bacterial pathogenesis with a resolution and precision that is not readily accessible using other model bacterial pathogens. Genetically encoded fluorescent reporters can be easily engineered into the T-DNA, which could allow using microscopy to noninvasively track the progression of infection with cellular resolution in single infected live plant cells^10^. Similar to *Agrobacterium*, viruses have been engineered to transfer reporter genes, allowing researchers to use microscopy to quantify specific molecular aspects of viral pathogenesis in single host cells^11,12^. Contrasting these measurements with predictions made by mathematical models has revealed fundamental aspects of viral pathology, but so far, this approach has largely eluded the study of bacterial pathogens due to a lack of transferred genetic material for most bacterial systems.

Here, we take advantage of T-DNA encoded reporters to engage in a back and forth between theory and quantitative measurements to dissect *Agrobacterium* pathogenesis. Using agroinfiltration of tobacco as a model system, we demonstrate that antagonism and synergy between bacteria shape the outcome of infection and constrain the use of *Agrobacterium* for plant engineering. We explored the cellular and molecular basis of these interactions using a combination of approaches including genetic perturbations, mathematical modelling and T-DNA expression measurements at the tissue and single-cell levels. These findings inspired us to engineer a new method to overcome the unique constraints imposed by this system.

## Results and discussion

### Transformation does not follow a Poisson distribution

To motivate our quantitative dissection of agroinfiltration, we started by asking if transformation can be described as a random process. To ground our expectations we used a random probabilistic model based on the Poisson distribution. This model, proposed almost nine decades ago to describe infection of *Escherichia* *coli* by bacteriophages^13^ remains the go-to mathematical framework to describe pathogen infection at the single-cell level. In the context of agroinfiltration, the Poisson distribution describes the fraction of plant cells *P* that get transformed by at least one strain of bacteria as a function of OD_*i*_, the optical density of that strain,

1 $$P=1-{{\mathrm{e}}}^{-\alpha {{{\mathrm{OD}}}}_{i}}$$

where *α* is a proportionality constant related to the transformation efficiency per unit of bacterial density of that given strain (see Supplementary Information for details).

Equation (1) was recently tested by Carlson et al. who argued that it was sufficient to describe agroinfiltration in tobacco^10^. To test if these findings were generalizable, we challenged this model by testing two key assumptions. The first one assumes that pathogens infect host cells independently of one another; that is, the infection efficiency on a per bacterium basis is a constant, regardless of their total population density. Thus, the likelihood of a plant cell being transformed by a given strain is exclusively determined by the density of bacteria belonging to this strain and not that of other strains. Second, this model assumes that the probability of a host cell getting infected is homogeneous across time and cells. Whether or how many times a cell is infected before by any given strain does not affect the likelihood of it being infected again, and there are no differences in susceptibility among host cells.

**Fig. 1:**
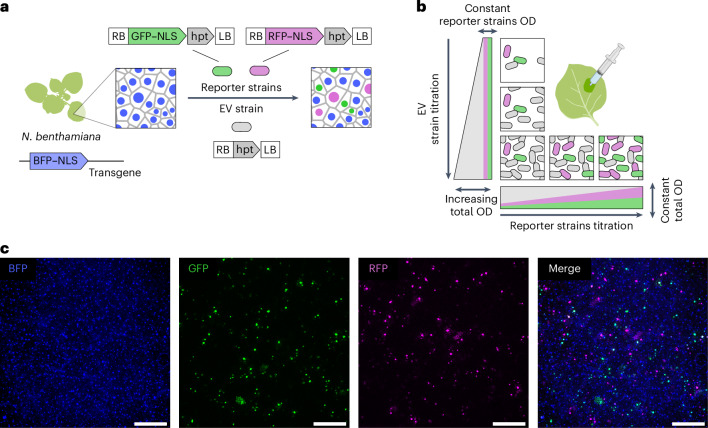
Experimental setup to quantify *Agrobacterium* T-DNA expression as a function of bacterial density and test the Poisson model of infection. **a**, A schematic of the experimental setup. A transgenic *N. benthamiana* line carrying a ubiquitously expressed nuclear-localized BFP–NLS transgene is used for agroinfiltration experiments. Three strains are mixed and infiltrated into the BFP–NLS line: two reporter strains carrying T-DNAs coding for a constitutively expressed nuclear-localized fluorescent proteins (GFP or RFP) and the hygromycin resistance hpt gene in the same T-DNA and an EV strain whose T-DNA codes only for hpt. RB, T-DNA right border; LB, T-DNA left border. **b**, A schematic of the reporter strain titration experiments. Along the vertical axis, the infiltration OD of the reporter strains is kept constant while the total culture OD is increased using the EV strain. Along the horizontal axis, the reporter strains are titrated while the total OD is kept constant using the EV strain. Both reporter strains, GFP and RFP, are mixed in equal ratios in each titration. Each mix is infiltrated into the leaves of the BFP tobacco line from **a**. **c**, The example maximum intensity projections of live fluorescence microscopy images obtained with the setup from **a** and **b**. Scale bar, 500 μm. **b** created with BioRender.com.

To quantify the number of infected plant cells, we used live fluorescence microscopy. We generated reporter *Agrobacterium* strains carrying binary vectors encoding plant fluorescent reporters in their T-DNA, either a green fluorescent protein (sfGFP) or red fluorescent protein (mCherry) fused to a nuclear localization signal and driven by a strong constitutive promoter (hereafter GFP and RFP reporter strains, respectively) (Fig. 1a). To experimentally control the total density of bacteria independent of the density of reporter strains, we created an empty vector strain (hereafter EV) with the same genetic background and carrying the same binary vector as reporter strains but without a fluorescent marker. To label host cells for counting, we generated a stable transgenic tobacco line with a constitutively expressed a nuclear-localized tagBFP2 blue fluorescent protein marker (hereafter BFP line) (Fig. 1a).

**Extended Data Fig. 1:**
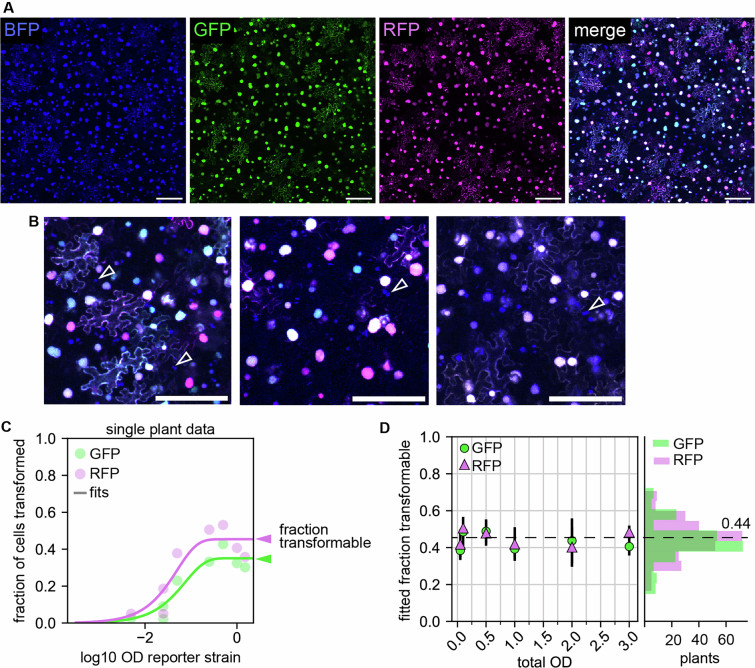
Estimation of the fraction of transformable cells. (**a**) Representative Maximum intensity projection images of a BFP tobacco leaf infiltrated with saturating ODs of GFP and RFP reporter cells (0.5 each). (**b**) details from the merged image in (**a**). Empty arrowheads indicate nuclei that do not express GFP nor RFP and are considered to be non-transformable by Agrobacterium in our setup. These nuclei are much smaller than other epidermis nuclei and tend to occur in pairs or clusters. (**c**) Example of the analysis used to obtain the fraction of transformable cells from images like the ones in (**a**). For each image the fraction of BFP nuclei that express GFP or RFP as a function of reporter strain OD was fitted to a sigmoidal function. The fraction at which the fits plateau is considered the ‘fraction of transformable cells’ of this image. (**d**) The fraction transformable was estimated as in (**c**) for all images. The plot on the left shows the mean ± SEM of this estimated fraction for GFP and RFP as a function of the total infiltration OD. The histogram on the left shows the distribution of the estimated values across the dataset. The mean of this distribution is used as the fraction of transformable cells throughout the article.

To experimentally obtain *P* as a function of OD_*i*_ and test equation (1), we titrated the reporter strains in equal ratios of OD (optical density at 600 nm) (Fig. 1b). We performed these titrations under different total bacterial densities, using the EV strain to reach increasing total bacterial ODs (Fig. 1b). These titration mixes were infiltrated into leaves of the BFP line, and a widefield microscope was used to obtain images of the leaf epidermis 3 days after infiltration. The blue channel was used to identify nuclei to then determine what fraction of these cells were infected by the reporter strains (Fig. 1c). Consistent with previous reports, we found that ~56% of epidermis cells are not susceptible to transformation^10,14^ (Extended Data Fig. 1). Hence, for the purpose of comparing the fraction of cells expressing RFP or GFP to the Poisson prediction, we use 44% of the total number of BFP nuclei as the total number of transformable cells, unless specified.

**Fig. 2:**
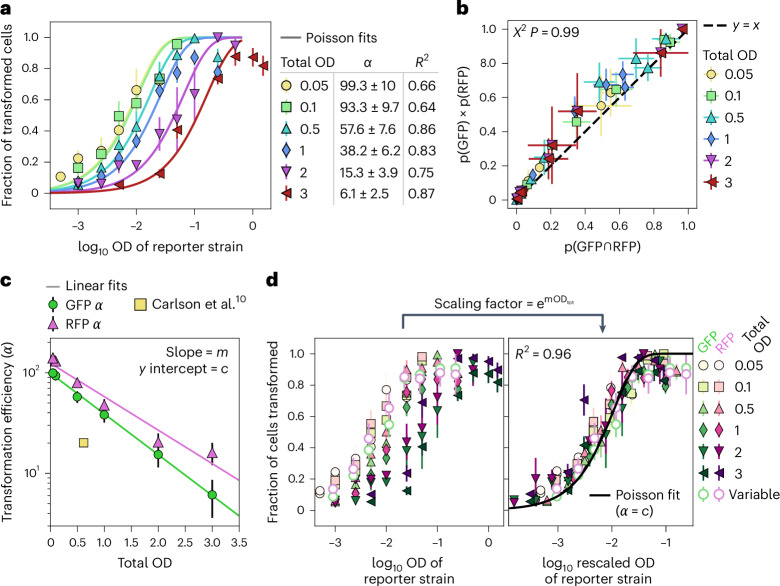
The transformation of plant cells by *Agrobacterium* does not follow a simple Poisson distribution due to antagonism. **a**, The mean ± standard deviation (s.d.) in the fraction of transformable nuclei expressing the GFP fluorescence reporter as a function of the infiltration OD of the GFP reporter strain for six infiltration mixes with increasing total ODs. The total ODs were achieved by adding EV cells as shown in Fig. 1. The solid curves show the best fit using equation (1) with *α* as the free parameter. The fitted *α* ± s.d. and the goodness of fit (*R*^2^) is shown (Extended Data Fig. 2). **b**, The expected mean fraction of nuclei coexpressing GFP and RFP if the two reporters are independent (*y* axis) is plotted against the mean observed fraction of nuclei expressing both GFP and RFP (*x* axis). The error bars are the s.d. across the images. The observed and expected values are not significantly different based on a chi-squared (*Χ*^2^) test (*P* value = 0.99). **c**, The *α* parameters obtained from the fits in **a** are plotted against the total infiltration mix OD. The error bars show the s.d. of the fits. The solid lines correspond to a linear fit to the log of *α* values. The *α* estimate from Carlson et al.^10^ is also shown. **d**, Left: the mean ± s.d. in the fraction of cells expressing GFP or RFP is plotted against the OD of that reporter strain. Right: the same as the left, except that the OD of the reporter strains was multiplied by a scaling factor ${{\mathrm{e}}}^{{{m{\mathrm{OD}}}}_{{{\mathrm{tot}}}}}$, where *m* is the slope of the linear fits in **c**, and OD_tot_ is the total OD of the infiltration mix. The ‘variable’ corresponds to infiltrations without EV. The solid line shows the Poisson prediction for *α* = *c*, where *c* is the *y* intercept of the linear fits in **c**. In **a**–**d**, *N* = 4, 7, 5, 8, 6 and 5 plants (one image per plant) for total ODs of 0.05–3, respectively. *N* = 5 plants for the variable OD data in **d**.

**Extended Data Fig. 2:**
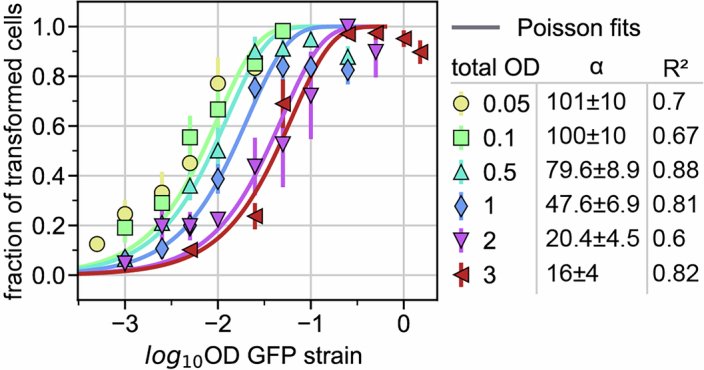
OD titration results for the RFP reporter strain. Shown is the mean ± SD in the fraction of nuclei detected in the RFP channel from the same set of experiments shown in Fig. 2a. As in Fig. 2a, the solid lines correspond to Poisson fits using Eq. 1. Also shown on the right are the parameter fits for ɑ and the goodness of fit (R^2^). N = 6 plants (one image per plant).

**Extended Data Fig. 3:**
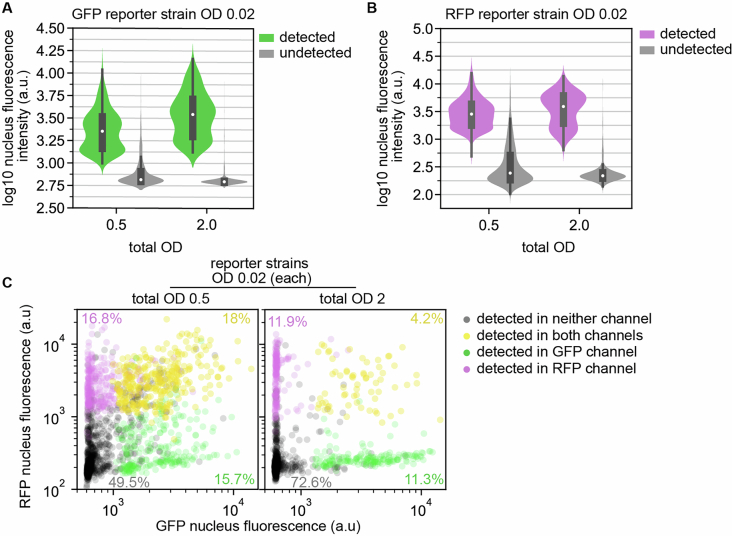
Differences in GFP and RFP fluorescence at the single nucleus level cannot explain why fewer nuclei are detected when EV is added at high ODs. The GFP and RFP reporter strains were infiltrated at OD 0.02 each with the addition of the EV strain at OD 0.46 or 1.96 for a final OD of 0.5 or 2.0. Images were acquired using confocal microscopy. (**a**) The pixel intensity in the GFP channel was calculated for each nucleus using BFP fluorescence to determine the nuclei volumes. In parallel, nuclei were classified as expressing GFP or not based on our image analysis pipeline. The violin plots show the fluorescence intensity in the green channel of nuclei classified as transformed by the GFP (green) or untransformed (gray). (**b**) Same as (**a**) but using the RFP channel. (**c**) scatter plots of single nucleus fluorescence in the green and red channels for all nuclei detected in the BFP channel. Each dot corresponds to a single nucleus and they are color-coded depending on whether they were assigned to the transformed or untransformed categories by our computational pipeline.

Comparing the fraction of BFP nuclei that express GFP as a function of the reporter strain OD shows that this data fits the Poisson model well (Fig. 2a). To test if the density of other strains affects the transformation efficiency by the GFP reporter strain, we titrated the EV strain to achieve increasing total culture ODs of 0.05, 0.1, 0.5, 1, 2 and 3. If the Poisson assumption that cell infection is a random and independent process is true, these curves should be identical regardless of total culture OD, that is, they should all be captured by a single form of equation (1). While at each total OD the curves fit equation (1) well (*R*^2^ = 0.66–0.87), these curves do not lie on top of each other (Fig. 2a). The same was true for RFP (Extended Data Fig. 2). At a given OD of the reporter strain, increasing the EV OD greatly reduces the fraction of transformed plant cells. As a result, the fitted transformation efficiency constant *α* decreases from ~100 for a total OD of 0.05 to ~6 for a total OD of 3 (Fig. 2a). To confirm that this finding is not a detection artefact due to lower detection rates at high ODs, we measured the fluorescence in the GFP channel of all nuclei, independent of whether they were assigned to the GFP^+^ or GFP^−^ categories by our computational pipeline. Nuclei detected as GFP^+^ have a fluorescence distribution that barely overlaps with that of those labelled as GFP^−^. Moreover, the green fluorescence distribution of GFP^+^ nuclei is largely identical at a total OD of 0.5 and 2.0, and the same is true for GFP^−^ nuclei (Extended Data Fig. 3). In addition, we imaged a subset of the samples in a laser scanning confocal microscope using slower but more sensitive settings. We found the results to correlate very well between machines (*R*^2^ = 0.87–0.91) (Supplementary Fig. 1). Thus, although agroinfiltration at a given total OD can be described as a Poisson process, the efficiency of transformation is not a constant but rather is inversely related to the total density of bacteria. This implies that infiltrated bacteria antagonize each other’s transformation efficiency, contradicting one of the key assumptions of the Poisson model previously postulated to describe agroinfiltration^10^.

Next, we tested the second assumption, namely, that all plant cells are equally susceptible and that whether a cell was transformed by one reporter strain does not affect its likelihood of being transformed by another. To this end, we measured the frequency of cells coexpressing GFP and RFP, p(GFP ∩ RFP), and compared it with its expected value if the two reporter strains are independent of each other, p(GFP) × p(RFP). Plotting these two frequencies against one another shows that they are very similar across total ODs (Fig. 2b). Although there is a small bias towards fewer cotransformed cells than expected if independent, we found that the independence hypothesis holds well (*X*^2^, *P* = 0.99). Hence, although the probability of transformation by a given strain decreases by increasing the OD of other strains, at a given total OD, transformation can still be considered random.

### A modified model of transformation incorporating antagonism

Our findings suggest that antagonism constitutes a ‘hidden variable’ in the model which scales down the transformation efficiency of each individual bacterium as a function of the total culture OD. This motivated us to test if incorporating the total OD as a variable into equation (1) could be used to correct the model for antagonism. We first plotted the transformation efficiency constant *α* as a function of the total OD and found that *α* and the total OD follow a simple exponentially decaying relationship with a negative slope *m* and a *y* intercept *c* (Fig. 2c). We took advantage of this simple relationship to obtain an antagonism correction parameter, ${{\mathrm{e}}}^{{{m{\mathrm{OD}}}}_{{{\mathrm{tot}}}}}$, where OD_tot_ is the total bacterial OD. When the infiltration OD of reporter strains is multiplied by this correction factor, it is possible to obtain their ‘effective OD’, OD_eff_. Using this effective OD allows us to collapse all the disparate curves from different total ODs into a single ‘master’ Poisson curve given by

2 $$P=1-{{\mathrm{e}}}^{-\alpha {{{\mathrm{OD}}}}_{{{\mathrm{eff}}}}},$$

where ${{{\mathrm{OD}}}}_{{{\mathrm{eff}}}}={{{\mathrm{OD}}}}_{i}\times {{\mathrm{e}}}^{{{m{\mathrm{OD}}}}_{{{\mathrm{tot}}}}}$. This curve represents transformation in the absence of antagonism, and consequently, its *α* is equal to *c*, the value of the transformation efficiency constant at an arbitrarily low total bacterial density. To show that this correction factor is generalizable, we titrated the reporter strains in the absence of the EV strain, that is, without keeping the total OD constant. These data also fall on the master curve (Fig. 2d). Thus, by taking the total bacterial density into account, it is possible to correct for antagonism and obtain a unique model that explains all our data. This correction factor suggests the presence of a simple underlying mechanism by which the effective infiltration ODs of reporter strains is scaled down due to antagonism.

We note that the *α* values previously reported using a single total OD of 0.6 do not lie on the curve of *α* as a function of OD reported here^10^ (Fig. 2c). Therefore, the use of different total ODs cannot explain the discrepancy between previous estimates of *α* and ours^10^. Because of the exponential dependence of the Poisson probability on *α*, these discrepancies have a large impact in applications that require expressing a single transgene per cell such as expressing pooled *trans*-acting gene libraries (Supplementary Fig. 2). For that kind of experiment, we recommend an OD of 0.0025.

### Investigating the basis of antagonism

**Fig. 3:**
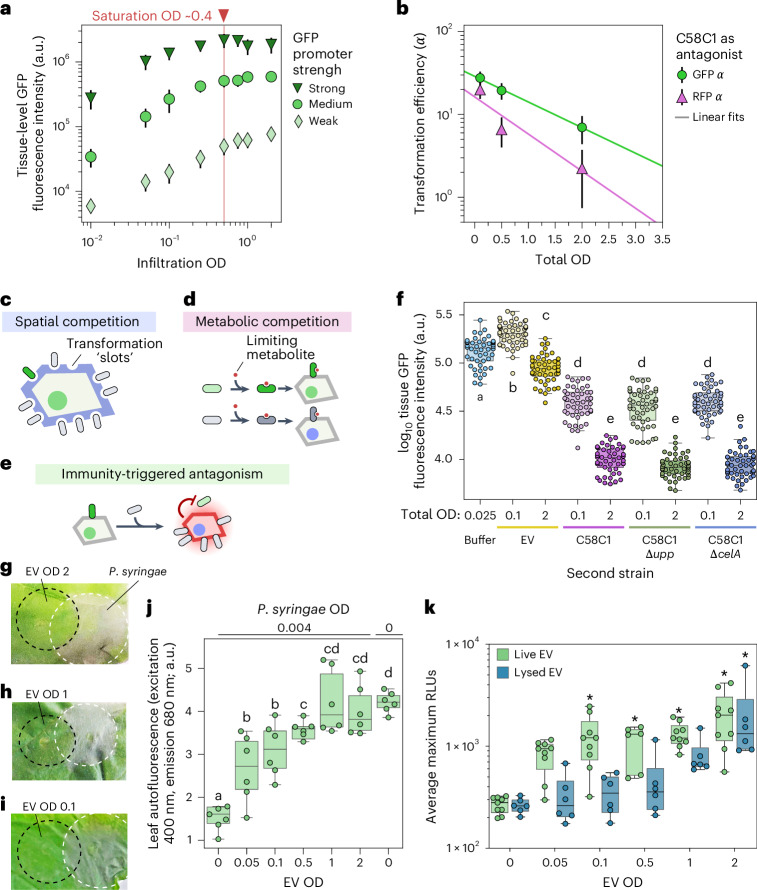
Antagonism takes place upstream of T-DNA delivery and correlates with an immune response from the host. **a**, The mean ± standard deviation (s.d.) tissue-level GFP fluorescence of leaves infiltrated with strains carrying a GFP transgene under promoters of different strengths. *N* = 48 (six plants with eight measurements per plant). **b**, The same as in Fig. 2c, using C58C1 instead of EV. The *α* parameters obtained from the Poisson fits are plotted against the total infiltration mix OD. The error bars show the fit s.d. *N* = 5 plants, one image per plant for each total OD. **c**, A cartoon of the spatial competition model. **d**, A cartoon of the metabolite competition model. **e**, A cartoon of the immunity-triggered antagonism model. High loads of the antagonistic strain trigger the immune system, which inhibits reporter bacteria. **f**, Box plots showing the leaf GFP fluorescence driven by a pCM2–GFP reporter strain infiltrated at a constant OD of 0.025. This reporter strain was combined with an antagonizing strain at an OD of 0.075 or 1.975 to achieve a final total OD of 0.1 and 2.0, respectively. A buffer was used as a control. *N* = 48 (six plants with eight measurements per plant). **g**–**i**, The representative images of leaves infiltrated with EV at increasing ODs (dashed black circles) and later infiltrated with *P. syringae* (white dashed circles). **j**, The tissue integrity measured using chlorophyll autofluorescence of leaves primed against *P. syringae* using EV infiltrated at different ODs as in **g**–**i**. The leaf punches were taken from the area overlapping both infiltrations. *N* = 6 plants with one leaf punch per plant. **k**, An immune response to *Agrobacterium* EV cells and EV lysates measured by ROS production. The relative fluorescence units (RLUs) include an average of four punches per plant, at least six plants per treatment. The asterisk denotes the statistical significance (*P* < 0.05) compared with water based on a post hoc Dunn’s multiple comparison test. In the box plots in **f**, **j** and **k**, the boxes show the median and the interquartile range. The whiskers show the minima and maxima excluding outliers, if present. In **f** and **j**, the letters on top of box plots indicate statistically significant (*P* < 0.05) groups based on a post hoc Tukey honestly significant difference test.

We reasoned that a decrease in transformation efficiency (that is, antagonism) should also manifest when a single reporter strain is titrated alone since there should be diminishing returns in T-DNA expression with increasing OD, and there should be an OD threshold above which there is no further increase in reporter expression. We titrated a GFP reporter strain alone and observed tissue-level saturation of GFP expression at OD of ~0.4 using three different promoters spanning two orders of magnitude in strength to drive the reporter^15^ (Fig. 3a). The fact that all three reporters saturate at a similar OD—albeit at different levels of GFP fluorescence—indicates that saturation is not due to the exhaustion of plant gene expression resources.

**Extended Data Fig. 4:**
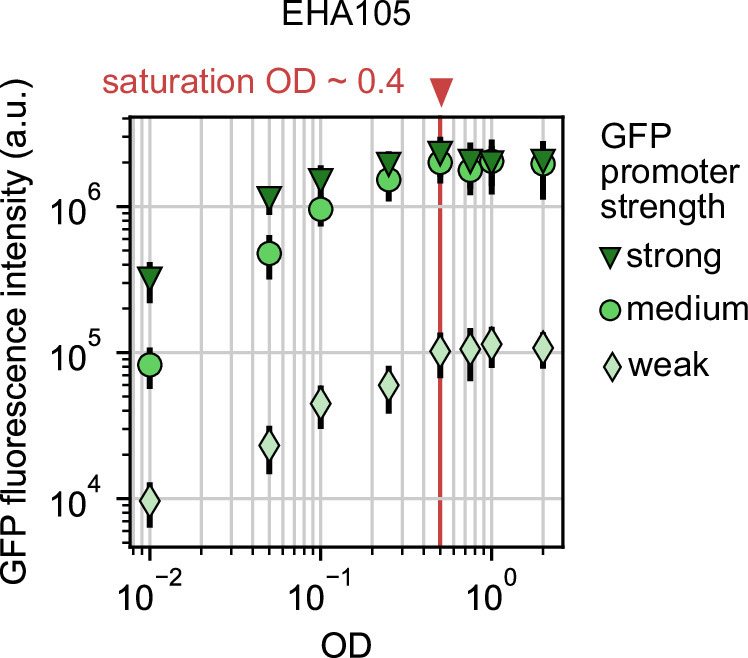
Tissue-level fluorescence driven by GFP reporter strains of the EHA105 background. As in Fig. 3a, shown is the mean ± SD of the tissue-level GFP fluorescence of agroinfiltrated leaves measured with a plate reader N = 48 (6 plants, 8 leaf punches per plant). The reporter strains belong to the EHA105 background and carry a GFP transgene under the control of three promoters of different strengths. The vertical red line indicates approximately at what OD the fluorescence saturates.

In principle, antagonism between bacteria could occur at two levels, upstream or downstream of T-DNA delivery. To test if T-DNA-related resources become limited with increasing OD, we repeated the GFP reporter strain titration using strain EHA105, which is derived from the supervirulent strain A281 (ref. ^16^). Tissue-level fluorescence saturated at a similar OD, consistent with antagonism being independent of pathogenesis (Extended Data Fig. 4). To further demonstrate that antagonism occurs upstream of T-DNA delivery, we repeated the single-cell imaging experiments shown in Fig. 2 using the strain C58C1 as the antagonist strain instead of EV. C58C1 is very closely related to GV3101 but lacks pTi and is unable to infect plant cells. As for EV, the transformation efficiency of reporter strains captured by the *α* parameter decreases exponentially with increasing C58C1 OD, showing that this strain is also capable of antagonizing the reporter strains (Fig. 3b and Supplementary Fig. 3). Taken together, these results demonstrate that antagonism occurs upstream of transformation, among bacteria in the apoplastic space outside the plant cell.

These data prompted us to explore the basis of apoplastic antagonism. We considered three non-mutually exclusive scenarios. First, since there is some evidence that *Agrobacterium* must attach to the plant cell wall to establish a pathogenic interaction, we speculated that antagonism could arise from competition for available area on the plant cell surface^17,18^ (Fig. 3c). Alternatively, bacteria may need to consume a plant metabolite to survive and/or mount an infection inside the leaf and this molecule may become limiting at high bacterial densities (Fig. 3d). Finally, antagonism might be of an indirect nature, arising from high bacterial loads triggering an immune response from the host that diminishes the transformation efficiency of each bacterium in a density-dependent manner (Fig. 3e). To rapidly determine the antagonistic interactions of multiple combinations of strains and test these models, we used a fluorescence plate reader assay, which is less laborious than live cell imaging. To assay for antagonism we measured the tissue-level fluorescence of leaves infiltrated with a GFP reporter strain at a constant OD of 0.025 in combination with different antagonizing strains at an OD of 0.075 (low) or 1.975 (high) to yield a final bacterial OD of 0.1 or 2.0. Here, a decrease in GFP fluorescence between high and low OD is used as a metric for antagonism.

**Extended Data Fig. 5:**
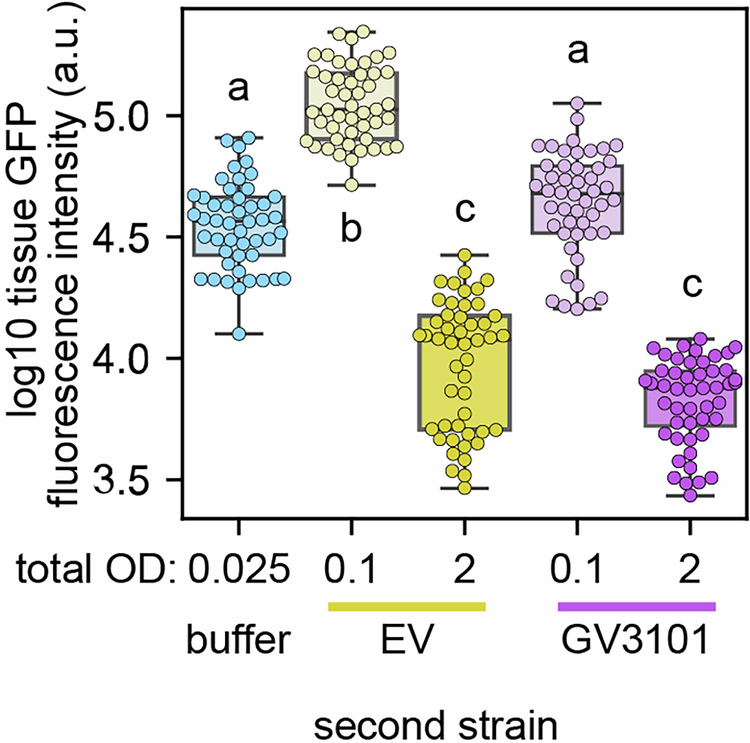
Synergy and antagonism driven by GV3101 at low and high OD, respectively. Box plots show the leaf tissue GFP fluorescence of N = 6 plants, 8 leaf punches per plant. As in Fig. 3e, a reporter strain carrying the medium strength pCM2-GFP transgene in its T-DNA was infiltrated at a constant OD of 0.025, either alone (‘buffer’) or in combination with a second strain (EV or GV3101) at low (0.075) or high (0.1975) OD. N = 48 (6 plants, 8 punches per plant). Boxes show median and interquartile range. Whiskers are minima and maxima excluding outliers. Letters indicate statistically significant (p < 0.05) groups based on a post-hoc Tukey HSD test.

**Extended Data Fig. 6:**
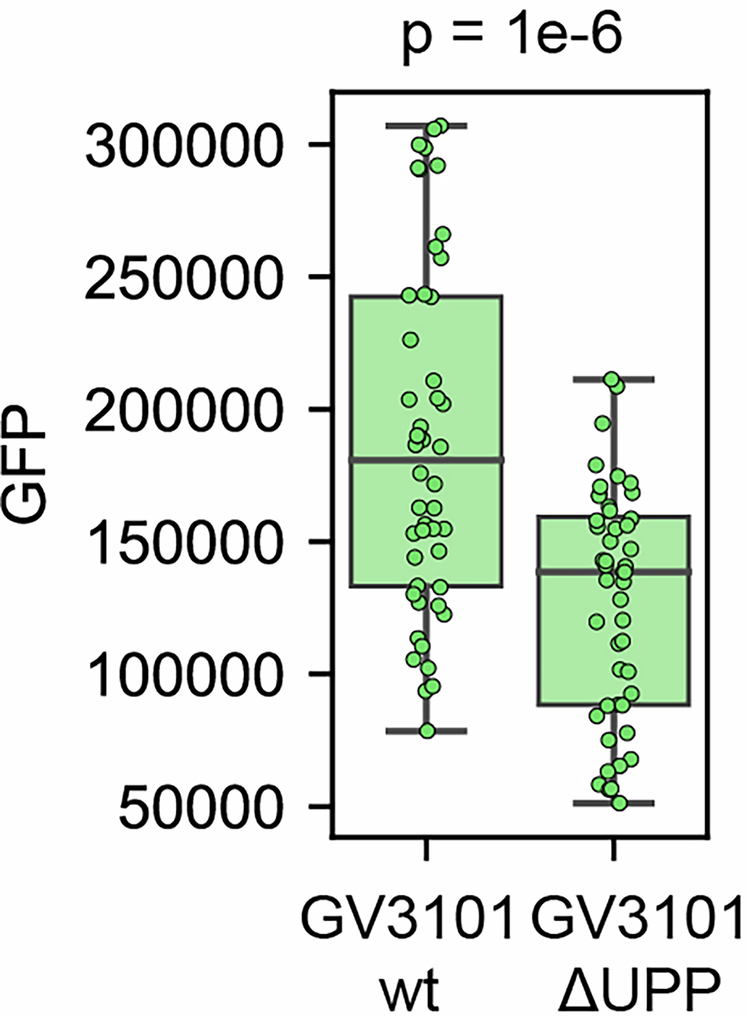
The formation of *Agrobacterium* attachment structures is required for efficient transformation during tobacco agroinfiltration. Shown is the tissue GFP fluorescence driven by reporter strains carrying a pCM2-GFP transgene in their T-DNA and infiltrated under identical conditions (OD 0.1). The wt GV3101 background strain is more efficient at transforming plant cells than the ΔUPP strain, which is unable to form the unipolar polysaccharide attachment structure. P-value of a two-sided Student’s t-test. Boxes show median and interquartile range. Whiskers are minima and maxima excluding outliers. N = 48 (6 plants, 8 leaf punches per plant).

To test the role of spatial competition, we impaired the attachment capacity of the antagonizing strain via genetic deletions. *Agrobacterium* attachment depends on multiple mechanisms, some of which require pTi^17,18^. As mentioned earlier, strain C58C1 (which lacks pTi) is still able to antagonize in our microscopy assay. We confirmed this result using the fluorescence plate reader assay (Fig. 3f). Strain GV3101, which carries pTi but not a T-DNA behaved similar to C58C1 under these conditions (Extended Data Fig. 5). We therefore used the C58C1 background to create knockouts in two genes necessary for the synthesis of polymers required for attachment: unipolar polysaccharide and cellulose (Supplementary Table 2). The antagonistic capacity of these mutants was indistinguishable from that of C58C1 (Fig. 3f), even though the attachment mutants exhibit reduced T-DNA expression in *N. benthamiana* (Extended Data Fig. 6). This suggests that competition for limited area on the plant cell surface is not a big contributor to antagonism. Intriguingly, we found a synergistic effect where adding EV but not C58C1 at a low OD resulted in higher GFP fluorescence compared with a buffer control (Fig. 3f).

To test the role of metabolic competition, we tested if supplementation with amino acids, trace minerals or vitamins could increase the level of GFP fluorescence driven by a reporter strain, either alone or in combination with C58C1 as an antagonist. None of the metabolites tested enhanced reporter expression under any of the conditions examined (Supplementary Fig. 4). These data suggest that apoplastic antagonism is not due to metabolic competition. However, these negative results cannot rule out that bacteria compete for apoplastic metabolites under other circumstances.

**Extended Data Fig. 7:**
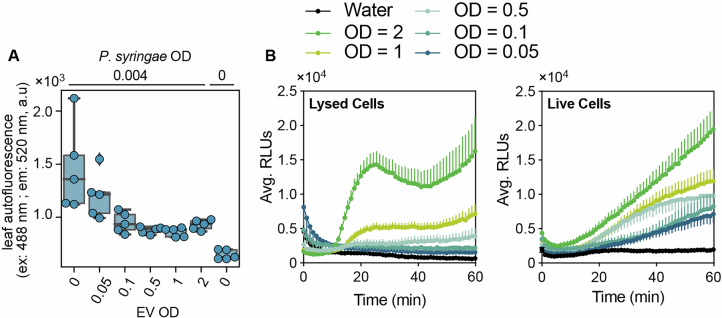
Immune response to Agrobacterium. (**a**) Same experimental setup as in Fig. 3g–j. Tissue integrity was determined using green autofluorescence. Boxes show median and interquartile range. Whiskers are minima and maxima excluding outliers. (**b**) Immune induction via ROS burst by increasing concentrations of Agrobacterium. Data from the same experiments shown in Fig. 3k. Error bars represent SEM based on at least six plants with four punches per plant. Average maximum RLU per plant was used in 3 K.

Next we focused on the potential effects of plant immune induction by *Agrobacterium*. Although *Agrobacterium* does not typically trigger a hypersensitive response in *N. benthamiana*, this host can recognize *Agrobacterium* epitopes^19–22^. However, this response is thought to be very weak in young (1 month old) plants such as the ones used in this study^23,24^. To test OD-dependent *Agrobacterium*-triggered immunity, we used an immune priming assay^25^. We infiltrated increasing ODs of the EV strain to elicit an immune response and tested to what extent the infiltrated tissue became immunoprotected against a subsequent exposure to *Pseudomonas syringae* pv. *syringae* B728a, which triggers cell death in tobacco (Methods). Stronger immune perception of *Agrobacterium* in this assay would prime the plant immune system against a secondary infection, reducing or eliminating subsequent cell death from a *P. syringae* infiltration^22^. We observed a clear OD-dependent response to *Agrobacterium* as measured by cell-death-dependent autofluorescence in primed tissue (Fig. 3g–j and Extended Data Fig. 7). This response saturates between OD 0.1–0.5, similar to our plate reader fluorescence assay (Fig. 3j).

To validate this finding and to better understand the nature of this immune response, we conducted a luminescence assay to detect reactive oxygen species (ROS) in tissues exposed to *Agrobacterium*. Extracellular ROS production is a hallmark of pattern-triggered immune recognition and can be used as a quantitative readout of this response. We challenged leaves with increasing densities of live or lysed EV bacteria and observed an OD-dependent ROS burst for both (Fig. 3k and Extended Data Fig. 7). Combined with our negative results regarding spatial and metabolic competition, these data are consistent with apoplastic antagonism among bacteria being indirect and stemming from the bacterial population as a whole triggering an immune response from the host. This response accentuates with increasing bacterial density and might impair the efficiency of transformation of individual bacteria by making plant cells more recalcitrant and/or by affecting bacterial fitness and survival. Studies in other plant species have shown that hindering the host immune response enhances *Agrobacterium* transformation^26,27^. In the future, disrupting the interaction between *Agrobacterium* epitopes and their receptors in *N. benthamiana* could provide an avenue for relieving OD-dependent antagonism.

### Synergistic T-DNA expression in strains carrying pTi

**Fig. 4:**
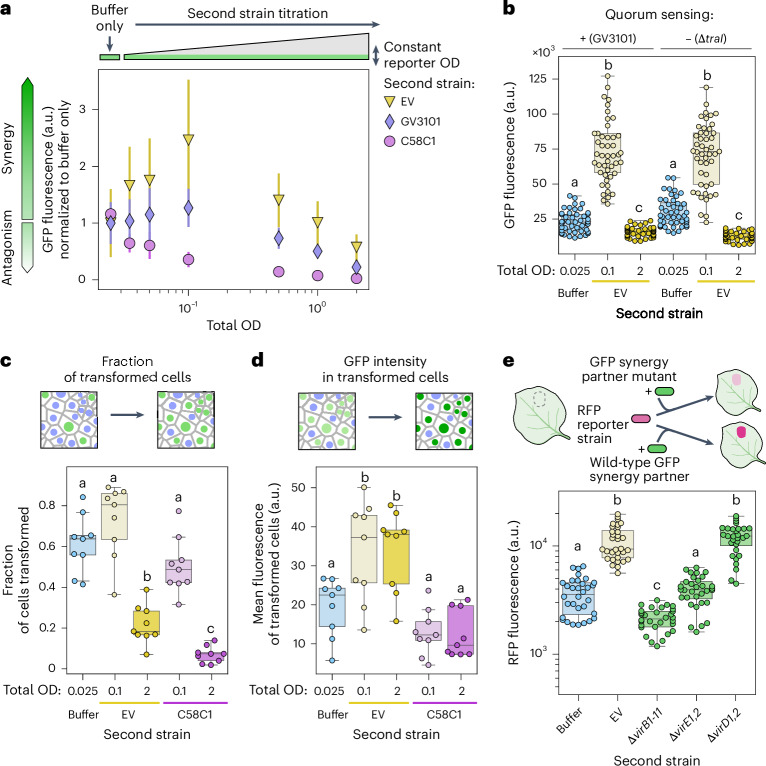
Synergistic T-DNA expression via sharing of secreted pTi-encoded effector vir proteins. **a**, The mean ± standard deviation fluorescence of leaves infiltrated with a GFP reporter strain at a constant OD of 0.025 alone (‘buffer only’) or combined with increasing amounts of a strain carrying pTi and a T-DNA (EV), pTi without a T-DNA (GV3101) or no pTi (C58C1). **b**, Quorum sensing is not required for synergy or antagonism. The GFP fluorescence of leaves infiltrated with a GFP reporter strain at an OD of 0.025 either alone (‘buffer’) or in combination with EV to achieve a total OD of 0.1 or 2.0. The background of both strains was wild-type GV3101 or quorum sensing defective Δ*traI*. **c**, Top: the possible cellular basis for the results in **a**, adding a second strain affects the fraction of cells transformed by the reporter strain. Bottom: the box plots of the fraction of nuclei expressing GFP in leaves infiltrated with a GFP reporter strain at a constant OD of 0.025 and a second strain added to achieve a total bacterial OD of 0.1 or 2.0. **d**, Top: the alternative cellular basis for the results in **a**, adding a second strain increases the fluorescence cells already transformed. Bottom: the box plots of the average GFP fluorescence across nuclei that were detected in the GFP channel in the experiments in **c**. In **c** and **d**, *N* = 9 plants, one image per plant. **e**, Top: a schematic of the experiment. An RFP reporter strain was infiltrated alone (OD 0.01) or in combination with a synergizing GFP reporter strain (OD 0.09). Bottom: box plots showing the leaf RFP fluorescence for different strain combinations. All strains have the GV3101 background, and *vir* mutants carry a binary vector with a GFP reporter. In **c**–**e**, the letters on top of box plots indicate statistically different groups (*P* < 0.05 based on a Tukey post hoc honestly significant difference test). In **a**, **b** and **e**, *N* = 48 (six plants with eight measurements per plant). The boxes in **b**–**e** are the median and interquartile range. The whiskers are minima and maxima excluding outliers. **e** created with BioRender.com.

As mentioned in the previous section, the tissue-level GFP fluorescence driven by a reporter strain can be increased by adding a low OD of EV cells (Fig. 3f and Extended Data Fig. 5). We refer to this phenomenon as bacterial synergy in T-DNA expression. Compared with the buffer control, adding C58C1 at a high or low OD reduced the tissue-level GFP fluorescence driven by the reporter strain. Addition of GV3101 reduced reporter expression at high OD but not at low OD (Extended Data Fig. 5). These data show that both the T-DNA and pTi are necessary for synergy and imply that pTi and the T-DNA mediate a density-dependent duality in *Agrobacterium* interactions. At low densities, strains carrying pTi can synergize with each other to achieve higher host expression of genes encoded in their T-DNA compared with each strain alone. With increasing ODs, antagonism between strains outweighs synergy and the total number of bacteria starts to negatively affect the transformation capacity of all strains (Fig. 3f). To test this duality, we performed a titration experiment where we kept the OD of the reporter strain constant at a low OD of 0.025 and added increasing ODs of a second strain carrying both pTi and a T-DNA (EV), just pTi (GV3101) or neither (C58C1). We then measured the tissue-level GFP fluorescence. When adding EV, the tissue-level GFP fluorescence shows a biphasic behaviour, progressively increasing with increasing OD but decreasing past an OD between 0.1 and 0.5 (Fig. 4a). We observed an attenuated form of the same behaviour using GV3101 (Fig. 4a). By contrast, coinfiltrating any amount of C58C1 cells results in a decrease in GFP fluorescence. Although the synergism by GV3101 may appear modest, comparing GV3101 with C58C1 reveals that synergy mediated by pTi in the absence of a T-DNA is sufficient to overcome antagonism at low ODs. The fact that at high OD the level of inhibition by EV is much weaker than by C58C1 suggests that synergism is also occurring at higher OD but is exceeded by the stronger antagonism at this OD. This shows how the T-DNA and pTi combined determine a density-dependent duality in bacterial interactions and highlights the importance of being able to deliver a T-DNA at a population level to achieve a robust infection (Fig. 4a). Importantly, ΔTraI strains, which are unable to produce the *Agrobacterium* quorum sensing signalling molecule, engage in synergy and antagonism to a similar extent than the wild type (Fig. 4b).

To narrow down the source of T-DNA and pTi-dependent synergy, we first focused on its cellular basis. An increase in tissue-level leaf fluorescence can result from two non-mutually exclusive underlying factors: an increase in the fraction of transformed cells or an increase in the level of fluorescence in these cells. We used a GFP reporter strain to measure both aspects in the presence of EV or C58C1 added at a low or high OD. As expected, at a high OD, addition of a second strain drastically reduces the fraction of GFP^+^ cells (Fig. 4c). However, at a low OD, neither EV nor C58C1 significantly changed the fraction of GFP^+^ cells compared with the buffer alone (Fig. 4c). Thus, the synergistic effect does not stem from an increase in transformation efficiency of the GFP reporter strain. We next measured the average nuclear fluorescence intensity exclusively among GFP^+^ nuclei. Here, EV is synergistic at low OD but not antagonistic at high OD (Fig. 4d). On the other hand, C58C1 was antagonistic at high OD and unable to synergize at low OD. These data demonstrate that synergy mediated by pTi and the T-DNA arises from an increase in T-DNA expression in cells that have already been transformed by the GFP reporter strain, not from an enhanced probability of transformation.

**Extended Data Fig. 8:**
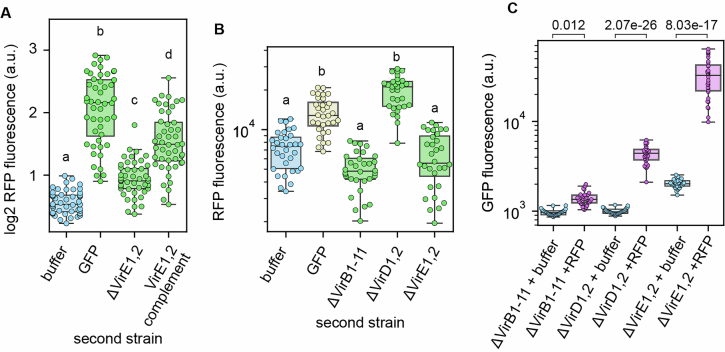
Role of vir genes in mediating synergistic effects. Boxplots showing tissue-level fluorescence. The experiments shown in (**a**) and (**b**) were conducted using the same setup as in Fig. 4e. Namely, a GV3101 RFP reporter strain was infiltrated alone at OD 0.01 (labeled ‘buffer’) or in combination with a second GV3101 GFP reporter strain carrying gene deletions in vir genes (OD 0.09). (**a**) The reduced synergy observed in ΔvirE1,2 can be partially restored by a complementation transgene. (**b**) Same as Fig. 4e but using pCM2-mScarlet as the transgene in the RFP reporter strain T-DNA instead of pUBQ10-mCherry. (**c**) Complementation of vir gene deletion knockouts by the RFP reporter strain. The same vir deletion strains carrying a GFP reporter T-DNA from (**b**) and Fig. 4e were infiltrated alone or in combination with the pCM2-mScarlet reporter strain from (**b**) and the tissue GFP fluorescence was measured. Compared to the buffer control, an increase in GFP fluorescence in the presence of the RFP strain indicates extracellular complementation of the vir mutant strain. In (**a**–**c**) N = 6 plants with 8 leaf punches per plant. In (A) and (B) letters correspond to statistically significant (p < 0.05) groups based on a post-hoc Tukey HSD test. In (**c**) p-value of a two-sided Student’s t-test. Boxes show median and interquartile range. Whiskers are minima and maxima excluding outliers. In (**a**–**c**) N = 32 (4 plants, 8 punches per plant).

*Agrobacterium* infection depends on the delivery of pTi-encoded vir effector proteins that are translocated into the host cell^28^. This led us to hypothesize that a strain may benefit from vir proteins delivered from a second strain, which may facilitate T-DNA transport into nucleus or increase T-DNA gene expression, resulting in higher GFP expression in transformed cells. To test if sharing effector proteins underlies synergy, we asked if tissue-level expression driven by an RFP reporter strain could be enhanced by previously generated GFP reporter strains carrying deletions in effector vir genes^29^. We also tested a Δ*virB1-11* GFP reporter strain, which lacks the type IV secretion system through which all effectors and the T-DNA are delivered. As shown in Fig. 4e, the synergy is completely lost in Δ*virB1-11*. Similarly, the Δ*virE12* strain cannot synergize with the reporter RFP strain (Fig. 4e). This decrease in synergy can be partially restored by a *virE12* complementation transgene (Extended Data Fig. 8). We also tested a Δ*virD12* mutant. Because VirD1 acts intracellularly and VirD2 is covalently attached to the T-DNA in its cell of origin, we thought it unlikely that this mutant would be impaired in synergy. Consistent with this, the synergistic effect of Δ*virD12* was comparable with that of EV (Fig. 4e). We obtained comparable results using a reporter strain carrying a different RFP reporter (Extended Data Fig. 8). These results show that secretion of effectors in general—VirE2 in particular—is necessary for synergy. Given that synergism by GV3101 is weaker than EV, it is possible that a functional T-DNA and/or sequences contained in the binary vector and not in pTi are required to efficiently produce or secrete VirE2.

These results are reminiscent of reports of ‘extracellular complementation’ of bacteria lacking secreted effector proteins such as VirE2 (refs. ^30–32^). We were also able to detect this kind of complementation of vir knockouts using reporter assays (Extended Data Fig. 8). However, the synergy we describe here occurs between strains that carry a full set of effector genes and are thus fully capable of transformation on their own. A similar phenomenon is the ‘helper effect’, where different wild type strains of *Agrobacterium* can enhance each other’s pathogenicity upon coinfection^33^. Yet, unlike the helper effect, the synergistic interactions that we have described here occur between pairs of wild-type background isogenic bacteria that differ only in the reporter gene. It will be interesting to understand how vir gene secretion interacts with cell density to give rise to a population behaviour that does not require quorum sensing (Fig. 4b).

### Strains harbouring two plasmids bypass antagonistic constraints

**Extended Data Fig. 9:**
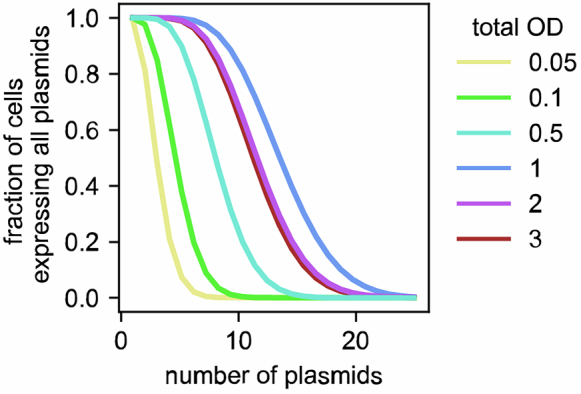
Effects of antagonism on the number of plant cells coexpressing multiple plasmids delivered by individual strains. The antagonism-adjusted Poisson model was used to predict the fraction of transformable plant cells transformed by all strains in a mix (y-axis) as a function of the number of plasmids, assuming 1 plasmid = 1 strain (x-axis). Different lines correspond to the prediction results for different total mix ODs. Note that increasing the mix OD past 1.0 (dark blue line) is predicted to decrease the fraction of plant cells coexpressing all T-DNAs. Hence, this model predicts that there is a hard limit to how much cotransformation can be increased by increasing the infiltration OD.

What are the implications of synergy and antagonism in an applied context? In plant metabolic engineering, candidate enzymes are often individually genetically delivered to plant cells by different *Agrobacterium* strains, allowing researchers to mix and match strains to rapidly iterate through the design–build–test cycle of pathway discovery^4^. To maximize the number of plant cells that express all these different transgenes, it is necessary to increase the total OD of the bacterial mix. However, our results show that this strategy has diminishing returns because the transformation probability of any one strain decreases exponentially by increasing the total OD of the mix. Hence, antagonism may constrain the diversity of transgenes that can be coexpressed per cell (Extended Data Fig. 9; see Supplementary Information for model details).

**Fig. 5:**
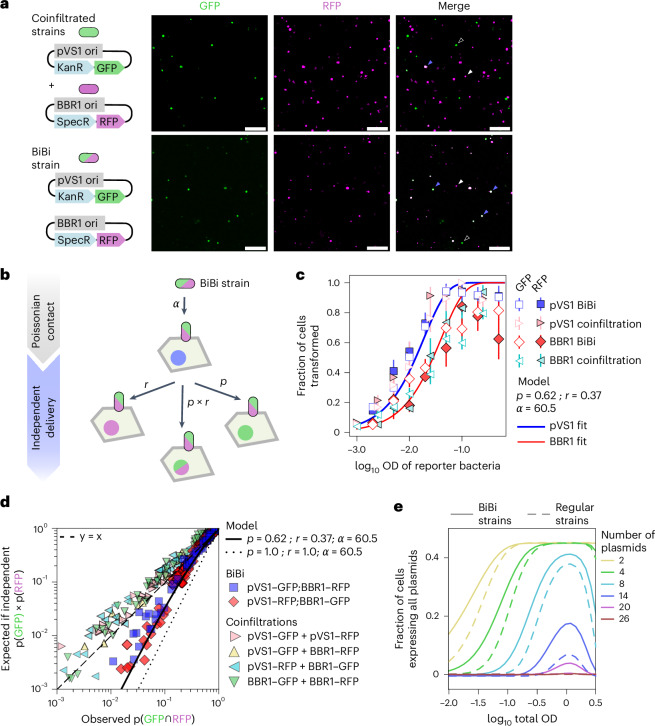
Characterization of plasmid codelivery in BiBi strains carrying two binary vectors per cell shows increased transgene expression diversity per unit of OD. **a**, Fluorescence images of tobacco leaves infiltrated with reporter bacteria at OD 0.002 and EV at an OD of 0.498. Top: reporter bacteria belonging to two strains, GFP in the pVS1 plasmid and RFP in the BBR1 plasmid, each at OD 0.001. Bottom: a single BiBi reporter strain at OD 0.002 carrying GFP in pVS1 and RFP in BBR1. The blue arrowheads highlight nuclei expressing both fluorescent proteins. The white and black arrowheads indicate nuclei expressing only RFP or GFP, respectively. Scale bar, 500 µm. **b**, A cartoon of the two-step BiBi codelivery model. Following Poisson-like contact, a bacterium can transfer the pVS1 plasmid with a probability *p* and the BBR1 plasmid with a probability *r*. Since plasmids are assumed to be independent, the codelivery probability is *p* × *r*. **c**, The mean ± standard deviation in the fraction of epidermis cells expressing GFP or RFP as a function of reporter bacteria infiltration OD. The total OD was kept constant at 0.5 using EV. *N* = 6 plants (one image per plant). The solid lines show the best fit of the model in **b** using the same set of values of *ɑ*, *p* and *r*. **d**, The expected fraction of cells coexpressing GFP and RFP if delivery of these transgenes is independent plotted as a function of the observed fraction of cells coexpressing both reporters. The dashed line shows *y* = *x*. The solid line shows the best fit of the model in **b** to the BiBi data using the same parameter values as in **c**. The dotted line shows the model prediction if *p* = *r* = 1.0. A single parameter set was used to fit the data in **c**, **d** and Supplementary Fig. 6 with a goodness of fit *R*^2^ = 0.53. **e**, The theoretical prediction of the fraction of cells coexpressing *N* plasmids if these plasmids are harboured by *N* regular strains (dashed lines) or *N*/2 BiBi strains (solid lines) for different total OD of the bacterial mix.

Since antagonism depends on the load of bacteria, to bypass this constraint we sought a way to increase the diversity of expressed T-DNAs without having to increase the culture density. To this end, we engineered strains carrying two binary vectors per cell, which we call ‘BiBi’ strains. One of the plasmid confers Kanamycin resistance and carries the pVS1 origin of replication while the other plasmid confers spectinomycin resistance and has the BBR1 origin. Hereafter, we refer to these binary vectors as pVS1 and BBR1. Although *Agrobacterium* strains carrying two binary vectors have been described before^34^, their performance at the level of single plant cells remains uncharacterized. To measure codelivery of different T-DNAs from BiBi strains, we infiltrated at a very low OD of 0.002 a BiBi strain carrying GFP in the BBR1 plasmid and RFP in the pVS1 plasmid. We compared this experiment to a coinfiltration of two regular strains, each one carrying either one of these vectors. The same OD of reporter bacteria was used in both cases (that is OD of GFP + RFP = OD BiBi). A visual inspection of these images revealed that the BiBi infiltration resulted in a much higher proportion of nuclei expressing both GFP and RFP compared with the coinfiltration control, demonstrating codelivery of T-DNAs from the pVS1 and BBR1 plasmids (Fig. 5a). However, nuclei expressing only one of the fluorescent proteins were also present in the BiBi infiltration (Fig. 5a), showing that the two BiBi T-DNAs are not always coexpressed.

The finding that different T-DNAs launched from BiBi strains are often but not always coexpressed in the same plant cell motivated us to develop a mathematical model to better understand this process. We abstracted T-DNA expression into two-steps (Fig. 5b). First, a bacterium has to make contact with the plant cell, which given our previous findings we assume to be Poisson-like with a probability constant *α*. Next, this bacterium can deliver either T-DNA, and this step has a probability of *p* for the pVS1 T-DNA and *r* for the BBR1 T-DNA. In this model, different T-DNAs harboured in the same BiBi strain are delivered independently of each other. That is, the probability of delivery of a given plasmid is the same regardless of whether it shares the same cell with a different plasmid. Hence, in a BiBi strain, the probability of coexpressing the pVS1 and BBR1 T-DNAs is *p* × *r* (Fig. 5b). To test if this simple model captures transformation by BiBi strains, we asked if we could use a single set of values of *p*, *r* and *α* to fit various aspects of BiBi microscopy data (see Supplementary Information for model details).

To test if the two BiBi T-DNAs are delivered independently, we measured the fraction of nuclei expressing GFP as a function of reporter bacteria OD when the GFP transgene is launched from the pVS1 or the BBR1 vector, with a second vector carrying RFP in the same BiBi strain or in a different coinfiltrated regular strain (Fig. 5c). Consistent with our model, the expression of pVS1-encoded GFP is largely independent of whether BBR1 RFP is launched from the same strain or a different coinfiltrated strain (Fig. 5c). Analogous results were obtained for BBR1, albeit the rate of transformation was lower for this binary vector compared with pVS1, implying that *r* < *p* (Fig. 5c). Intriguingly, when two BBR1 strains are coinfiltrated alone, their rate of transformation increases and becomes similar to that of pVS1 (Supplementary Fig. 5). To further validate our model and constrain the parameter fits, we plotted the fraction of nuclei expressing both GFP and RFP versus its expected value given independence (as in Fig. 2b). The coexpression of T-DNAs delivered by BiBi strains was far more frequent than expected if they were independent (Fig. 5d). Yet, the BiBi codelivery frequency is lower than the expectation if pVS1 and BBR1 were always codelivered, given by *p* = *r* = 1 (Fig. 5d). To further challenge our model, we used other single-cell aspects of the data (Supplementary Fig. 6). We found that all these disparate observations can be well explained by our model with parameters *p* = 0.62 ± 0.034, *r* = 0.37 ± 0.03 and *α* = 60.15 ± 5.4 (overall *R*^2^ = 0.53 for all the combined data shown in Fig. 5 and Supplementary Fig. 6).

These results demonstrate that BiBi strains increase the fraction of cells that express multiple T-DNAs per unit of OD. To understand the impact of the BiBi system in a more realistic scenario with a large number of transgenes, we combined equation (2) with the BiBi model of Fig. 5b. We calculated the fraction of leaf cells that express *N* plasmids when these plasmids are delivered by *N* regular strains or *N*/2 BiBi strains at different total ODs (Fig. 5e). According to this prediction, packing *N* plasmids in *N*/2 BiBi strains always leads to a higher fraction of cells expressing *N* transgenes, although this increase is small to negligible when *N* is low. For *N* ≥ 14, coexpression using regular strains becomes extremely unlikely but is considerably increased using BiBi strains (see Supplementary Fig. 7 and Supplementary Information for model details).

### BiBi strains for complex metabolic engineering

**Fig. 6:**
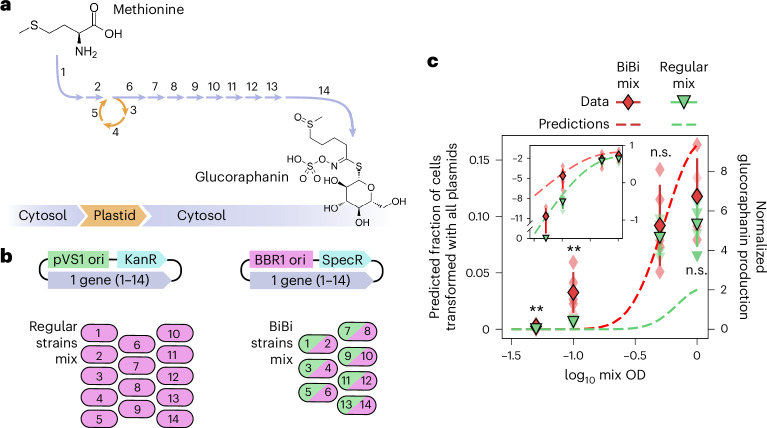
Testing the implications of *Agrobacterium* antagonism for long engineered biosynthetic pathways. **a**, An overview of the glucoraphanin biosynthesis reconstitution experiment in tobacco. 14 transgenes need to be transiently expressed via agroinfiltration for the efficient biosynthesis of glucoraphanin from endogenous methionine as a precursor in tobacco leaves. These transgenes encode for 13 enzymes and one plastid transporter. All reactions occur in the cytosol, except for the ones catalysed by enzymes 3, 4 and 5, which are plastidial. **b**, Each of the 14 genes in **a** was cloned individually in a pVS1 and a BBR1 plasmid. These plasmids were then used to create 14 regular BBR1 strains and 7 BiBi strains. In BiBi strains, the even-numbered genes from **a** are carried by pVS1 and the odd-numbered in BBR1. **c**, The two mixes from **b** were infiltrated into tobacco leaves at four different total ODs, 0.05, 0.1, 0.5 and 2.0, and glucoraphanin production was quantified. The mean ± standard deviation of normalized glucoraphanin concentration (right *y* axis) and the predicted fraction of cells coexpressing all 14 transgenes (left *y* axis) as a function of the infiltration OD is shown. The red diamonds correspond to BiBi infiltrations, and the green triangles correspond to regular BBR1 strains. The dashed line shows the prediction from Fig. 5e, assuming that the fraction of cells expressing the 14 transgenes is linearly proportional to glucoraphanin production. The inset shows the data and predictions using the same *x* axis and *y* axis in a log_10_ scale. The asterisks indicate the result of a two-tailed *t*-test. n.s., not significant, **P value ≤0.01, *N* = 5 plants.

We next sought to understand to what extent bacterial antagonism limits the reconstitution of complex biosynthetic pathways and whether this constraint can be overcome by the BiBi system. We focused on the 14-gene biosynthetic pathway of the chemopreventive natural product glucoraphanin, which has previously been reconstituted in tobacco^35^ (Fig. 6a). We cloned each of these genes in individual BBR1 vectors to create a mix of 14 strains. To test BiBis, we cloned the odd-numbered genes from the glucoraphanin pathway in the pVS1 vector and combined them in consecutive order with the even-numbered genes cloned in the BBR1 vector to obtain a mix of 7 BiBi strains (Fig. 6b and Supplemental Table 1).

To ground our expectations for the performance of the different mixes, we took advantage of our theoretical models based on fluorescent protein reporters. We based our prediction on the assumption that the amount of glucoraphanin produced is linearly related to the fraction of plant cells that coexpress all 14 genes (Fig. 5e). According to this model, the difference between regular and BiBi strains should be more pronounced at low mix ODs (Supplementary Fig. 7). Hence, to fully explore the benefit of using BiBi over regular strains, we infiltrated each of the mixes at four different total ODs: 0.05, 0.1, 0.5 and 1.0. Liquid chromatography–mass spectrometry (LC–MS) was then used to quantify glucoraphanin production in infiltrated leaves.

**Extended Data Fig. 10:**
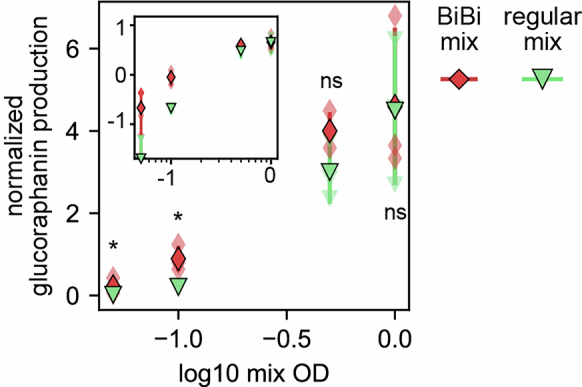
Replicate of the glucoraphanin biosynthesis experiment shown in Fig. 6c. The two mixes from Fig. 6b were infiltrated into tobacco leaves at 4 different total ODs, 0.05, 0.1, 0.5, and 2.0 and glucoraphanin production was quantified. Shown is the mean ± standard deviation of normalized glucoraphanin concentration. Red diamonds correspond to BiBi infiltrations and green triangles correspond to regular BBR1 strains. Asterisks indicate the result of a two-tailed t-test where ns indicates not significant and * indicates a p-value ≤ 0.05 with N = 3 plants. Inset: data and predictions using the same x-axis and the y-axis in a log10 scale.

At an infiltration OD of 0.05 glucoraphanin was detected in the BiBi mix but not in the mix of 14 regular strains. At an OD of 0.1, a product was detected in both infiltrations but the concentration in the BiBi infiltration was about an order of magnitude higher. Thus, under these conditions, delivering the 14 transgenes in 7 BiBi strains can increase product formation compared with 14 single-plasmid strains (Fig. 6c). This finding is largely consistent with our theoretical prediction (Fig. 6c). This level of agreement strongly suggests that the improvement of BiBi over regular strains is due to a larger proportion of plant cells coexpressing all transgenes, as seen for fluorescent proteins. At ODs 0.5 and 1.0, glucoraphanin production was slightly higher for BiBi than regular strains. Although this difference was not found to be statistically significant, it was reproducibly observed in a separate independent replicate of the whole experiment (Extended Data Fig. 10). This difference at high ODs was smaller than the prediction, a discrepancy that most probably stems from nonlinear effects associated with metabolic pathways that are not at play when using fluorescent protein reporters (Fig. 6c). Most saliently, metabolic intermediates may diffuse between adjacent plant cells via plasmodesmata^36^, allowing intercellular complementation when most cells express most T-DNAs. Overall, the agreement between our theoretical predictions and the data at low ODs indicates that, by increasing the fraction of cells coexpressing all T-DNAs, BiBi strains may provide an advantage for longer pathways or in scenarios where it is not desirable or possible to use high ODs.

## Conclusion

For nearly four decades, *Agrobacterium*-mediated gene transfer has been the cornerstone of plant biotechnology, yet the scrutiny of this process at a cellular level has remained qualitative. More recently, tobacco agroinfiltration has enabled a number of methods at the cutting edge of plant biology such as massively parallel reporter assays, synthetic circuit design and elaborate metabolic engineering. Such complex applications are bound to grow in popularity, scope and sophistication, demanding a quantitative and predictive understanding of T-DNA expression in single plant cells. Here, we develop the most comprehensive framework so far to model transgene expression at the cellular level.

This dialogue between predictions and experiments allowed us to discover a number of previously underappreciated aspects of *Agrobacterium* pathogenesis such as apoplastic antagonism, synergistic T-DNA expression and two-step stochastic T-DNA delivery in BiBi strains. Conceptually, these phenomena are not limited to *Agrobacterium* and reveal principles applicable to other bacterial pathogens. Given the biotechnological importance of *Agrobacterium*, these findings have clear implications for applied engineering efforts, as demonstrated by the use of BiBi strains to increase the diversity of coexpressed transgenes.

## Methods

### Plasmids and *Agrobacterium* strains

All plasmids used in this study can be accessed from the JBEI public repository (https://public-registry.jbei.org/). The BFP–NLS plasmid was created in a previous study^37^. The binary vectors used for the GFP, RFP and the EV strain were based on the pCambia1300 backbone. The BBR1 plasmids were custom made based on the pGingerBS backbone^38^. All plasmids were created using standard Gibson assembly or Golden Gate assembly protocols. All plasmids were transformed into the *Agrobacterium* of the GV3101::pMP90 background via electroporation. In frame, internal gene deletions were constructed via allelic exchange in strains C58C1 or GV3101 as described previously^29^. A list of all the plasmids used in this study along with their link to the JBEI registry can be found in Supplementary Table 2.

### Plant growth conditions

*N. benthamiana* (tobacco) plants were grown in an indoor growth room kept at 60% humidity and 25 C temperature under a 16–8 h light–dark daily cycle and 120 μmol m^−2^ s^−1^ of light intensity. The plants were grown in Sunshine Mix no. 4 soil (Sungro) supplemented with 499 Osmocote 14-14-14 fertilizer (ICL) at 5 ml l^−1^ and infiltrated with *Agrobacterium* cultures 29 days after sowing.

### *Agrobacterium* growth conditions

*Agrobacterium* glycerol stocks were streaked on Luria–Bertani (LB) plates containing antibiotics. The day before infiltration, single colonies were grown overnight in liquid LB containing antibiotics, shaking at 30 °C. The day of infiltration, the cultures were diluted 1:10 in LB with the same antibiotic concentrations and grown for a few more hours under the same conditions until reaching an OD_600_ of 0.5–1.0. The antibiotic concentrations used for all strains except C58C1, GV3101 and strains carrying BBR1 plasmids were: 50 μg ml^−1^ rifampicin, 50 μg ml^−1^ kanamycin and 30 μg ml^−1^ gentamicin. The strains carrying BBR1 plasmids were grown using 50 μg ml^−1^ rifampicin, 100 μg ml^−1^ spectinomycin and 30 μg ml^−1^ gentamicin. GV3101 was grown in 50 μg ml^−1^ rifampicin and 30 μg ml^−1^ gentamicin. C58C1 was grown without antibiotics.

### Agroinfiltration

*Agrobacterium* liquid cultures at an OD_600_ of 0.5–1.0 were spun down at 4,000*g* for ~10 min and resuspended in a similar volume of infiltration buffer (10 mM 2-(*N*-morpholino)ethanesulfonic acid pH 5.6, 10 mM MgCl_2_, 150 μM acetosyringone). The cultures were incubated in infiltration buffer shaking for 1 h at room temperature before the OD_600_ measurements. Next, a 1:5 dilution of each culture in the infiltration buffer was prepared, and the OD_600_ of this dilution was measured using a spectrophotometer. The final OD dilutions for infiltration were then prepared using infiltration buffer. The mixes were infiltrated in the sixth and seventh leaf (counting upwards with cotyledons being leaves 1 and 2) within 1 h from preparation. The position within the leaf of each infiltration was randomized between plants.

### Imaging, widefield fluorescence microscopy

All widefield fluorescence images were taken in a Leica DM6B microscope. Three sequential images were taken for each *z*-stack, one for each fluorescent protein. The filter sets used were: the 4,6-diamidino-2-phenylindole filter for BFP (excitation 350 ± 50, emission 460 ± 50), the L5 filter for GFP (excitation 480 ± 40, emission 527 ± 30) and the Texas Red filter for RFP (excitation 560 ± 20, emission 630 ± 38). A 5× dry objective was used to acquire 2.641 mm^2^ images of 2,048 × 2,048 pixels for a pixel size of 0.78 µm^2^. Five *z*-sections were acquired every 20 µm. The laser power was set to 17% for all channels. The camera exposure time was 800 ms. All confocal images were acquired using a Zeiss LSM710 microscope. In each *z*-slice, two sequential scans were used, one for BFP and RFP and another one for GFP. In the first scan, excitation wavelengths were 405 nm using the diode and 568 nm using the InTune laser. The two emission windows in this first scan were 410–530 nm (for BFP) and 585–630 nm (for RFP). The second scan used the argon 488 nm laser for excitation and an emission window of 494–581 nm for GFP. The frame size was 2,048 × 2,048 pixels with 1.5 zoom using a 5× dry objective, resulting in a 1.133-mm^2^ image with a pixel size of 0.55 µm^2^. The scans were performed bidirectionally at a speed set to 9, corresponding to a pixel dwell time of 0.39 µs and a scan time of 7.75 s per slice. The averaging during acquisition was done using two lines. The laser intensities and gain settings were chosen to avoid detector saturation and were as follows. The laser power was 1.0 for 405 nm, 4.0 for 568 nm and 1.0 for 488 nm, and gain was 711 for BFP, 589 for RFP and 480 for GFP. The pinhole was set to 1 a.u. in both scans. The *z*-slices were taken every 2.5 µm, with enough slices to capture all the epidermis nuclei in the field of view. The cell fluorescence intensity data shown in the same graphs comes from images taken during the same imaging session to avoid day-to-day variation in laser power.

### Image analysis, nuclei segmentation

The max-intensity *z*-projections of each channel were generated and a difference of Gaussians (DoG) filter with a sigma of five pixels was applied. The DoG images were then segmented into objects (that is, nuclei) and background using a fluorescence threshold value. To find this fluorescence value, we used the Otsu method included in the skimage Python package. The result of this thresholding is a binary image of nuclei and background. Each of these binary images was visually inspected and compared side by side to the original max projection to check the segmentation by eye. The fluorescence threshold applied to the corresponding DoG image was adjusted whenever necessary. We used the watershed algorithm to separate nuclei that merged with each other. Finally, an area filter was applied to the binary nuclei image mask to remove objects with an area smaller than 40 pixels, which for circular objects corresponds to ~3.5 pixels in radius or ~2.5 µm. As a reference, the guard cell nuclei (the smallest nuclei in the epidermis) have a radius of ~4 µm. We also filtered out objects with an area larger than 900 pixels.

### Image analysis, calculating the fraction of transformed nuclei

The total number of nuclei used as the denominator to calculate fractions corresponds to the number of objects found in the BFP channel as described in the ‘Image analysis, nuclei segmentation’ section. To determine how many nuclei were found in the GFP channel, we generated a GFP mask and applied it to the BFP mask by multiplying these binary images. Next, we applied an area filter to the resulting image using the same area thresholds as for the BFP mask. We then counted the number of objects, which correspond to nuclei detected as expressing GFP. The same procedure was used for RFP. To determine if a nucleus expresses both GFP and RFP, we asked if the centroid of a given GFP-detected object overlaps that of an RFP-detected object. The objects whose centroids are closer than five pixels were counted as expressing both reporters.

### Image analysis, nucleus fluorescence intensity

The confocal *z*-stacks were segmented into binary images of nuclei and background as described in the ‘Image analysis, nuclei segmentation’ section. We applied an erosion algorithm to shave off boundary pixels from the edges of binary objects to ensure that only pixels that belong to the nucleus were included. Next, for each binary object, we calculated the mean fluorescence intensity of the pixels in the corresponding max-projected image which overlap with this binary object. For the purpose of this average, we did not include the 5% brightest and dimmest pixels so as to remove outlier values.

### Immune response assays, priming against *P. syringae* and measuring ROS burst

*N. benthamiana* bacteria-triggered ROS bursts were measured using a luminol-horseradish peroxidase assay as previously described^39^. Briefly, three 4-mm-diameter leaf punches were taken using a biopsy punch from four plants and floated on 150 µl of water overnight in darkness. The incubation water was then removed and a 100-µl water solution of L-012 (Wako Chemicals, cat. no. 143556-24-5), horseradish peroxidase (Sigma-Aldrich, cat. no. P6782), and differing OD of either live cells or dead cell lysates were added to each well containing a leaf disk. The lysates were collected similarly as described at five different optical densities: 0.05, 0.1, 0.5, 1 and 2 (ref. ^20^). The luminescence was measured immediately after adding the 100-µl solution using a Tecan Infinite F Plex every 1.1 min for at least 60 min. At least two independent experiments were performed. To measure *N. benthamiana* immune system priming by *A. tumefaciens* against *P. syringae*, an assay was conducted as previously described^25^. Briefly, five optical densities of EV cells (0.05, 0.1, 0.5, 1 and 2) along with a buffer negative control were infiltrated randomizing the leaf positions of each OD between plant replicates (‘Agroinfiltration’ section). A total of 7 h after infiltrating EV, fresh *P. syringae* cultures resuspended in agroinfiltration buffer at an OD of 0.02 were infiltrated in an area partially overlapping with the EV infiltration. This results in a leaf region containing both *A. tumefaciens* and *P. syringae* (where the infiltration areas overlap) as well as a region that contains only *P. syringae*. A total of 3 days post infiltration, the leaf punches were taken from the area where both bacterial infiltrations overlap and chlorophyll autofluorescence was measured using 450 nm excitation and 680 nm emission (‘Leaf fluorescence measurements using plate reader assays’ section).

### Glucoraphanin extraction

All *Agrobacterium* strains were grown as detailed in the ‘Agroinfiltration’ section. Before resuspending in infiltration buffer, the OD of each strain was measured and strains were mixed in equal amounts. The strains were then centrifuged at 4,000*g* for 20 min and resuspended in infiltration buffer. From this mix, infiltration mixes of different total OD were prepared. In the case of regular strains (BBR1 plasmid) the OD of each strain coding for each of the glucoraphanin biosynthesis enzymes was this total bacterial OD divided by 14. In the case of BiBi strains, the OD of each strain was this same total OD divided by 7. These different mixes were incubated shaking at room temperature for 2 h. The entire seventh leaf (with cotyledons counting as leaves 1 and 2) of *N. benthamiana* plants were syringe-infiltrated with the appropriate mixture in quintuplicate per OD condition. These plants were returned to the growth room (‘Plant growth conditions’). After 5 days, the infiltrated leaves were removed and deveined with a razor blade to remove all major veins. In addition, ~5 mm of tissue around the entire periphery of the leaf was removed. The tissue was then placed into a preweighed 2-ml tube and flash frozen in liquid N_2_. All the samples were then lyophilized for 3 days in a −45 °C lyophilizer. Following lyophilization, the tubes were weighed, and the total dried mass was calculated. Next, two 2-mm metal balls were added per tube, and the tissue was pulverized in a PowerLyzer at 2,000 rpm for 5 min. The tubes were spun down, and 10 µl of 80% MeOH containing 2.5 ppm of methionine sulphone as an internal normalizer were added per 1 mg of ground tissue. The tubes were returned to the PowerLyzer and allowed to bead beat at 2,000 rpm for 10 min to extract the glucoraphanin. The tubes were then spun at 20,000*g* for 10 min following this, and the supernatant was removed and transferred to a new tube. These tubes were then flash frozen in liquid N_2_ and returned to a chilled centrifuge to spin at 20,000*g* for 10 min to precipitate salts. Avoiding the pellet, the supernatant was then transferred to a 10 kDa filter column and spun for 30 min at 20,000*g*. This final crude extract was used for LC–MS quantification of glucoraphanin.

### LC–MS analysis

An LC–MS analysis of glucoraphanin was conducted on a Waters Acquity UPLC BEH Amide column (100-mm length, 2.1-mm internal diameter and 1.7-μm particle size; Waters Corporation) using a 1290 Infinity II ultra-high performance liquid chromatography (UHPLC) system (Agilent Technologies). A sample injection volume of 1 μl was used throughout. The sample tray and column compartment were set to 4 °C and 30 °C, respectively. The mobile phase was composed of 10 mM ammonium acetate, 0.2% ammonium hydroxide (Sigma-Aldrich) and 5 µM medronic acid (Sigma-Aldrich) in water (solvent A) and 10 mM ammonium acetate, 0.2% ammonium hydroxide (Sigma-Aldrich) and 5 µM medronic acid (Sigma-Aldrich) in 80% acetonitrile and 20% water (solvent B). The solvents were of LC–MS grade and were purchased from HoneyWell Burdick and Jackson. The analytes were eluted via the following gradient conditions: held at 100% B for 0.5 min, linearly decreased from 100% B to 75% B in 2.43 min, linearly decreased from 75% B to 70% B in 0.35 min, held at 70% B for 0.55 min, linearly decreased from 70% B to 50% B in 0.2 min, held at 50% B for 0.3 min, linearly increased to 100% B in 0.2 min and held at 100% B for 2.47 min. The flow rate was held at 0.36 ml min^−1^ for 4.33 min, linearly increased from 0.36 ml min^−1^ to 0.5 ml min^−1^ in 0.2 min and held at 0.5 ml min^−1^ for 2.47 min. The total UHPLC run time was 7 min.

The UHPLC system was coupled to an Agilent Technologies 6545 quadrupole time-of-flight mass spectrometer. The quadrupole time-of-flight mass spectrometer was tuned with Agilent Technologies ESI-L low concentration tuning mix (at a tenth of its concentration) in the range of 50–1,700 *m*/*z*. Electrospray ionization via the Agilent JetStream Source was conducted in the negative ion mode (for [M − H]^−^ ions) and a capillary voltage of 3,500 V was utilized. Drying and nebulizer gases were set to 10 l min^−1^ and 20 lb per square inch, respectively, and a drying-gas temperature of 300 °C was used throughout. The Agilent JetStream Source sheath gas temperature and sheath gas flow rate were set to 350 °C and 12 l min^−1^, respectively, while the nozzle voltage was set to 2,000 V. The fragmentor, skimmer and OCT 1 RF Vpp voltages were set to 100, 50 and 300 V, respectively. The data acquisition rate was set to 0.86 spectra per second. The data acquisition range was from 60 to 1,100 *m*/*z*. The data acquisition (Workstation B.08.00) and processing (Qualitative Analysis B.06.00 and Profinder B.08.00) were conducted via Agilent Technologies MassHunter software. The metabolites were quantified via external calibration curves, with *R*^2^ coefficients of ≥0.99.

### Leaf fluorescence measurements using plate reader assays

For tissue-level fluorescence measurements, four 6-mm leaf disks were cut from each agroinfiltrated leaf using a single-hole puncher and placed on top of 300 µl of tap water in a black, clear-bottom, 96-well plate. A BioTek Synergy H1 plate reader was then used to measure the GFP fluorescence of each leaf disk using an excitation wavelength of 488 nm and an emission wavelength of 520 nm. For RFP fluorescence, an excitation wavelength of 587 nm and an emission wavelength of 615 nm were used.

### Curve fitting and parameter inference

Curve fitting and parameter inference were performed via nonlinear least squares using the Python SciPy package. No parameter bounds were used for fitting anywhere in the text. The fit in Fig. 5c,d and Extended Data Fig. 6 was performed using all data shown in these figures simultaneously.

### Generation of transgenic BFP *N. benthamiana*

The transgenic benthamiana lines were produced at the Ralph M. Parsons Foundation Plant Transformation Facility at the University of California Davis using standard tissue culture methods. The strain *Agrobacterium* *fabrum* GV3101 carrying plasmid UtB2N7 (ref. ^37^) was used for the transformation.

### Material availability

The bacterial strains can be requested from the JBEI public registry by contacting JBEI strain archivists after signing an MTA agreement. The seeds are available upon request.

### Reporting summary

Further information on research design is available in the Nature Portfolio Reporting Summary linked to this article.

## Supplementary information

Supplementary Information Supplementary Text (calculations), Figs. 1–7 and Tables 1–3.

Reporting Summary

Supplementary Data 1 Processed microscopy data.

Supplementary Data 2 Plate reader fluorescence data.

Supplementary Data 3 Glucoraphanin LC–MS data.

## Data Availability

The nuclei counts from microscopy experiments, leaf punch fluorescence intensity from plate reader experiments and LC–MS data are available as Supplementary Tables. All microscopy raw data are available from the authors upon request.

## Extended data

are available for this paper at 10.1038/s41477-025-01996-w.

## Code availability

All the code used for data analysis is described via GitHub at https://github.com/shih-lab/agro_poisson.
