# Supplementary material for: Quantitative dissection of Agrobacterium T-DNA expression in single plant cells reveals density-dependent synergy and antagonism
Source: Nat Plants. 2025 May 12;11(5):1060–73. doi: 10.1038/s41477-025-01996-w (PMC12095076; doi:10.1038/s41477-025-01996-w)
Supplement: Supplementary file 1 — Supplementary Text (calculations), Figs. 1–7 and Tables 1–3. [file 41477_2025_1996_MOESM1_ESM.pdf]

nature plants

Supplementary information https://doi.org/10.1038/s41477-025-01996-w

Quantitative dissection of *Agrobacterium* T-DNA expression in single plant cells reveals density-dependent synergy and antagonism

In the format provided by the authors and unedited

Alamos et al. Quantitative dissection of Agrobacterium T-DNA expression in single plant cells reveals density-dependent synergy and antagonism.

Supplementary Text

S1 Details about the Poisson distribution applied to Agrobacterium-mediated transformation

Here we elaborate in more detail the motivations, assumptions and derivation of the Poisson model from Equation 1. The Poisson distribution P computes the probability of observing k number of occurrences of a random event within a discrete interval such as a span of time or space given a probability constant λ,

$$P(k, \lambda) = \frac{\lambda^k e^{-\lambda}}{k!} \tag{S1}$$

The λ constant is defined as the average number of occurrences across all intervals and is thus defined as

$$\lambda = \frac{\text{total number of ocurrences}}{\text{number of intervals}} \tag{S2}$$

In the context of a host-pathogen interaction, the intervals correspond to the host cells and the occurrence of a random event corresponds to infection by a single pathogenic agent. This approach to modeling infection allows us to predict what fraction of host cells are infected by zero, one, two, or any arbitrary number of pathogens given a value of λ. From the definition of λ presented above, it follows that applied to a pathogen interaction context,

$$\lambda = \frac{\text{total number of infection events}}{\text{number of host cells}}, \tag{S3}$$

where an infection event is defined as the contact of one host cell by a single bacterium, allowing for multiple bacteria to infect each cell. Because the total number of infection events is nearly impossible to determine, we make the assumption that this number is proportional to the number of bacteria, such that

$$\lambda \propto \frac{\text{total number of bacteria}}{\text{number of host cells}}, \tag{S4}$$

where $\propto$ means 'proportional to'. Notice that the ratio between the number of bacteria and the number of plant cells can be understood as the density of bacteria. We assume this density to be proportional to the OD$_{600}$ density of the culture infiltrated into the leaf, OD_i, meaning that

$$\lambda \propto OD_i. \tag{S5}$$

We now introduce the constant α to capture this proportionality. This means that by multiplying OD_i by α it is possible to obtain the value of λ. With this proportionality conversion at hand we can now rewrite Equation S1 as

$$P(k, \alpha, OD_i) = \frac{(\alpha OD_i)^k e^{-\alpha OD_i}}{k!} \tag{S6}$$

To compare the distribution predicted by Equation S6 with experiments it would be necessary to count what fraction of plant cells are transformed by $k = 0, 1, 2, ...$ number of bacteria. This is complicated by the fact that is not easy to distinguish a cell transformed by one bacterium from a cell transformed by 2 or more. However, we can use Equation

S6 to make predictions that can more easily be tested via experiments. Specifically, we can predict the fraction of cells that are transformed *at least once*, which is a much more accessible measurement. This is given by

$$P(k > 0) = 1 - P(k = 0) = 1 - e^{-\alpha OD_i}, \tag{S7}$$

Which is Equation 1 from the main text.

S2 Calculating the fraction of cells expressing all plasmids when launched from regular strains

Assuming that all N strains in a mix transform plant cells independently of one another, the probability that a plant cell gets infected by all strains is simply the multiplication of the probability of it being infected by each of them. This independence assumption is well supported by our own data (Figure 2B).

As in the main text, we use P for the Poisson probability and $P(k > 0)$ for the probability of being infected at least once by a given strain. Hence, for N strains, the probability of being infected at least once by all of them is

$$[P(k > 0)]^N \tag{S8}$$

Following our derivation in the main text (Equation 4), we know that

$$P(k > 0) = 1 - e^{-\alpha OD_{eff}}, \tag{S9}$$

where α is a constant to go from the effective infiltration OD of a given strain, OD_{eff} to a Poisson probability. In turn, as mentioned in the main text, the effective infiltration OD is given by

$$OD_{eff} = OD_i \times e^{mOD_{tot}} \tag{S10}$$

where OD_i is the infiltration OD of each strain in the mix, OD_{tot} is the total OD of the mix and m is a constant. If the mix is composed of N strains mixed in equal OD ratios then $OD_i = OD_{tot}/N$. Combining Equations S8-S10 we obtain our final formula for the fraction of cells coexpressing all N T-DNAs,

$$\text{fraction of cells expressing } N \text{ T-DNAs} = \left[1 - e^{-\alpha \times (OD_{tot}/N) \times e^{mOD_{tot}}}\right]^N \tag{S11}$$

From the linear fits in Figure 2C we estimate the values of the constants α and m to be approximately 100 and -0.9, respectively.

S3 Why the predicted fraction of cells cotransformed by multiple strains decreases at high ODs

Our model predicts that past a total OD of ~ 1 the efficiency of transformation by a mix of strains starts to decrease. This is somewhat counterintuitive, so here we explain how this results arises from the math. Specifically, how a scaling factor that incorporates the negative effect of OD_{tot} on the transformation probability can explain this result.

It is evident that, if we mix N strains in a total OD of OD_{tot}, the effective OD of each strain increases by increasing OD_{tot}. This much can be seen in the formula for the effective OD

$$OD_{eff} = OD_{tot}/N \times e^{mOD_{tot}}, \tag{S12}$$

where, ignoring the $e^{mOD_{tot}}$ correction factor, OD_{eff} scales linearly with OD_{tot}. What we need to understand is why at some point near a total OD of 1 the correction factor overcomes the positive effect of increasing OD_{tot} and makes OD_{eff} start to decrease. To get at this question, we calculate the first derivative of OD_{eff} with respect to OD_{tot} to then see what determines the sign of this derivative

$$\frac{dOD_{eff}}{dOD_{tot}} = \frac{e^{mOD_{tot}}(mOD_{tot}+1)}{N} \quad \text{(S13)}$$

Because N is a constant (the number of strains), it does not affect the sign of this derivative and can be ignored for our purposes. The term $e^{mOD_{tot}}$ is always positive regardless of the value of m or OD_{tot} so it cannot explain why OD_{eff} starts to decrease at high OD_{tot}. Finally, we see that, because m is negative and $\sim$ = -0.9, the term $mOD_{tot}+1$ becomes negative when $OD_{tot} > 1.11$.

Hence, when $OD_{tot} > 1.11$, the effective OD of each of the strains in a mix starts to decrease with increasing total mix OD. This implies that for the purposes of coexpressing multiple transgenes, there is nothing to gain from infiltrating past a total OD of about 1.1.

S4 Derivation of the plasmid co-delivery model in BiBi strains

The experimental motivation behind this model is the fact that BiBi strains (carrying two binary plasmids with distinguishable reporters) sometimes yield nuclei expressing only one of the reporters. We argue that this reflects the fact that a bacterium can establish a contact with a plant cell and yet fail to deliver one or both of the T-DNAs it carries.

To model this scenario quantitatively we make three assumptions. First, we assume that T-DNA transfer is a two-step process: a bacterium has to establish a contact with a plant cell and then it has to transfer the T-DNA. Note that by 'transfer' we are lumping together a number of steps, from DNA delivery to detectable expression. Second, we assume that the establishment of contacts occurs at random following a Poisson distribution with a probability that is proportional to the OD of the strain (Figure 2 and Equation 4 in the main text). Third, we assume that -given a contact- each bacterium has a finite probability of transferring a T-DNA.

We want to derive a mathematical expression for the probability of finding a plant nucleus expressing both T-DNAs. We start with the probability of nuclei expressing one of the T-DNAs, ignoring the second one for now. We'll call this T-DNA pVS1 in reference to the experiment where one of the binary vectors in the BiBi strains carries the pVS1 origin of replication. Let's define

$$\text{probability of transferring pVS1} = p, \quad \text{(S14)}$$

meaning that the probability of not transferring pVS1 is given by

$$\text{probability of not transferring pVS1} = 1 - p = q. \quad \text{(S15)}$$

Because we are assuming that the establishment of a contact and the transfer of the pVS1 T-DNA are independent of each other, the probability of a nuclei receiving $pVS1$ is the probability of a contact being established multiplied by the probability of transferring pVS1, p. Using P to denote the Poisson distribution we have that the probability of one contact leading to the transfer of pVS1 is

$$\text{probability of detecting pVS1 in one contact} = P(k=1) \times (1-q), \quad \text{(S16)}$$

where k is the number of contacts. The reason for using $1-q$ instead of its equivalent, p, will become clearer shortly. With increasing OD a plant cell can experience more than one *Agrobacterium* contact. Let's examine the 2 contact scenario. Given 2 contacts there are 4 possible outcomes:

**Probability table for 2 contacts**			
**contacts leading to T-DNAs transfer**	**1st contact**	**2nd contact**	**probability**
both	yes	yes	p^2
one	yes	no	pq
one	no	yes	pq
none	no	no	q^2

Using fluorescent protein reporters we can only say see whether at least one T-DNA gets expressed, not how many. Hence, what we are interested in is the probability that *at least one* of the contacts succeeds. From table the 2-contact tbale we see that the only outcome where none of the T-DNAs gets transferred has a probability of q^2. Therefore,

$$\text{probability of detecting } pVS1 \text{ in two contacts} = P(k=2) \times (1-q^2) \tag{S17}$$

What if there are three contacts? following the 2-contact case we can build the following probability table

**Probability table for 3 contacts**				
**T-DNAs transferred**	**1st contact**	**2nd contact**	**3rd contact**	**probability**
three T-DNAs	yes	yes	yes	p^3
two T-DNAs	yes	yes	no	p^2q
two T-DNAs	yes	no	yes	p^2q
two T-DNAs	no	yes	yes	p^2q
one T-DNA	yes	no	no	pq^2
one T-DNA	no	no	yes	pq^2
one T-DNA	no	yes	no	pq^2
zero T-DNA	no	no	no	q^3

In all scenarios except for the last one we would be able to detect this T-DNA. So the probability of detecting pVS1 in a three-contact scenario is the probability of obtaining three contacts (given by the Poisson probability of k=3) multiplied by one minus the probability of the zero-contact case, q^3

$$\text{probability of detecting pVS1 in three contacts} = P(k=3) \times (1-q^3). \tag{S18}$$

By now it is easy to see that, for a number of contacts k the probability of not detecting the T-DNA is $1-q^k$. For a given OD of the strain carrying $pVS1$ we can then calculate the overall probability of detecting the T-DNA by summing over all the possible number of contacts, namely,

$$\text{probability of detecting } pVS1 = \sum_{k=1}^{\infty} P(k) \times (1-q^k). \tag{S19}$$

Now we can introduce the second plasmid carrying the T-DNA $BBR1$. Let us define

$$\text{probability of transferring } BBR1 = r, \tag{S20}$$

and

$$\text{probability of not transferring the } BBR1 \text{ T-DNA} = 1-r = s. \tag{S21}$$

Note that the two T-DNAS, $pVS1$ and $BBR1$ are equivalent, their only difference being their respective probabilities of getting transferred. Further, in our model the two binary vectors do not care about the presence of each other, so we treat them as fully independent. With this in mind now we want to come up with a formula to calculate the probability of detecting both the $pVS1$ and the $BBR1$ T-DNAs in the same nucleus when delivered by a BiBi strain. Following our treatment for a single T-DNA, we can express the probability of one bacterial contact where both $pVS1$ and $BBR1$ get transferred as

$$\text{probability of detecting } pVS1 \text{ and } BBR1 \text{ in one contact} = P(k=1) \times (1-q) \times (1-s), \tag{S22}$$

where the Poisson probability of one contact, $P(k=1)$, and the probabilities that one $pVS1$ and one $BBR1$ are transferred multiply each other because they are mutually independent. In a k-contact scenario, we saw that the probability of transferring at least one $pVS1$ was $(1-q^k)$, and so the probability of transferring at least one $BBR1$ is $(1-s^k)$. Thus we can establish the overall probability across all numbers of contacts k as

$$\sum_{k=1}^{\infty}\left[P(k)\times(1-q^k)\times(1-s^k)\right]. \tag{S23}$$

The summation of a product is the product of the summations, so we can rewrite this expression as

$$\sum_{k=1}^{\infty}P(k)\times\sum_{k=1}^{\infty}\left[(1-q^k)\times(1-s^k)\right]. \tag{S24}$$

Since the first term in this product is the Poisson probability of more than zero contacts, we can rewrite as

$$P(k>0)\times\sum_{k=1}^{\infty}\left[(1-q^k)\times(1-s^k)\right]. \tag{S25}$$

We can expand this formula a bit to make it more explicit

$$(1-e^{\lambda})\times\sum_{k=1}^{\infty}\left[(1-q^k)\times(1-s^k)\right]. \tag{S26}$$

We saw in the main text that that λ is proportional to the OD of the strain times a proportionality constant, α. Hence, the probability of detecting $pVS1$ and $BBR1$ at a given OD of a BiBi strain OD_i is

$$(1-e^{\alpha OD_i})\times\sum_{k=1}^{\infty}\left[(1-q^k)\times(1-s^k)\right]. \tag{S27}$$

We used this formula to fit all the the experimental data in Figure 5C, D, and Figure S9 simultaneously.

S5 Calculating the fraction of cells expressing all plasmids when launched from BiBi strains

To calculate the fraction of cells expressing all plasmids from a mix of BiBi strains, we assume that strains make contacts with plant cells independent of each other. Hence, to calculate the probability of more than one strain transferring both its plasmids we simply multiply the probabilities of each strain transferring both plasmids (as in the previous section, Equation XX). If all BiBi strains are infiltrated at the same OD and all bacteria in the mix are BiBi strains, this corresponds to raising the probability of one strain to a power corresponding to the number of strains

$$\left[\sum_{k=1}^{\infty}\frac{(\alpha OD_{tot}/N)^k e^{-\alpha OD_{eff}}}{k!}\times(1-q^k)\times(1-s^k)\right]^N \tag{S28}$$

Where N is the number of strains such that OD_{tot}/N is the OD of each strain. As before, OD_{eff} is the 'effective' OD after correcting for competition by multiplying the infiltration OD of each strain by the scaling factor $e^{mOD_{tot}}$, where OD_{tot} is the total OD of the mix and m is a constant.

Supplementary Materials

Quantitative dissection of *Agrobacterium* T-DNA expression in single plant cells reveals density-dependent synergy and antagonism.

Simon Alamos, Matthew J. Szarzanowicz, Mitchell G. Thompson, Danielle M. Stevens, Liam D. Kirkpatrick, Amanda Dee, Hamreet Pannu, Ruoming Cui, Shuying Liu, Monikaben Nimavat, Ksenia Krasileva, Edward E. K. Baidoo, Patrick M. Shih.

Supplementary Figures

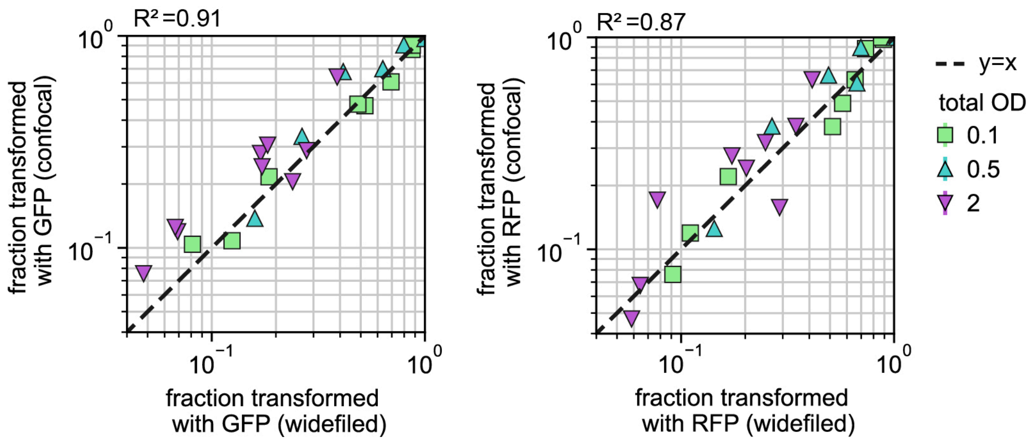

Supplementary Figure 1. Related to Figure 2: Imaging the same set of samples in a confocal setting with overall better resolution supports the accuracy of widefield microscopy. A subset of the samples used in Figure 2 were also imaged in a laser scanning confocal microscope right after acquiring the widefield fluorescence images. In the confocal experiments a higher magnification and better signal quality setting were used, at the expense of much slower data acquisition. The fraction of cells transformed by GFP or RFP was calculated using the same pipeline as for the widefield images. Finally, the results from both experiments were plotted against one another as scatter plots.

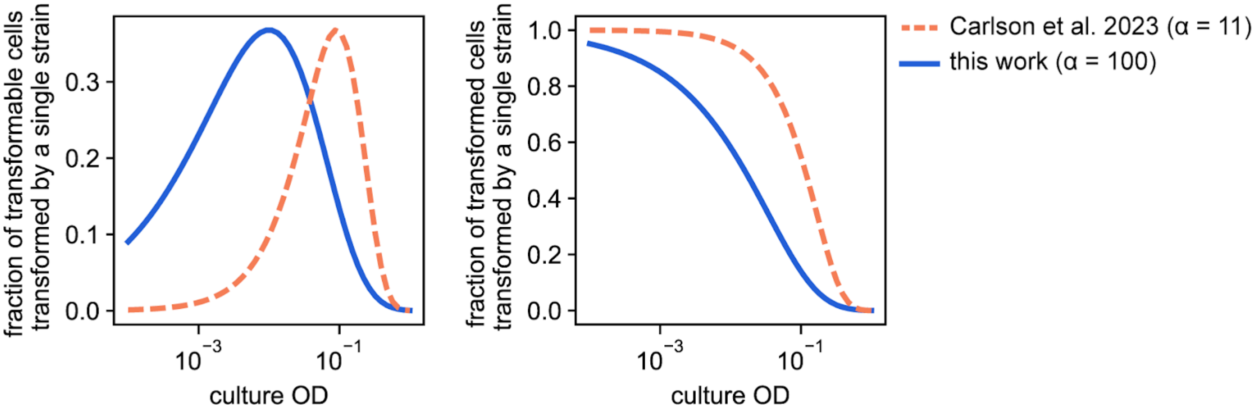

Supplementary Figure 2. Related to Figure 2: Contrasting Poisson predictions based on two different estimates of the transformation efficiency constant α. Left: Prediction of the fraction of transformable plant cells that are transformed by a single *Agrobacterium* cell as a function of the culture OD. Right: prediction of the fraction of plant cells that do get transformed that are transformed by a single *Agrobacterium* cell. Two values of the transformation efficiency constant α were used to generate both predictions. The dashed orange line uses α = 11 as estimated by Carlson et al. 2023 (10). The solid blue line uses α = 100 as estimated in this work. If the goal is to maximize the number of cells that are transformed by a single bacterium, these two estimates make starkly different recommendations. We recommend an OD of 0.002-0.0025.

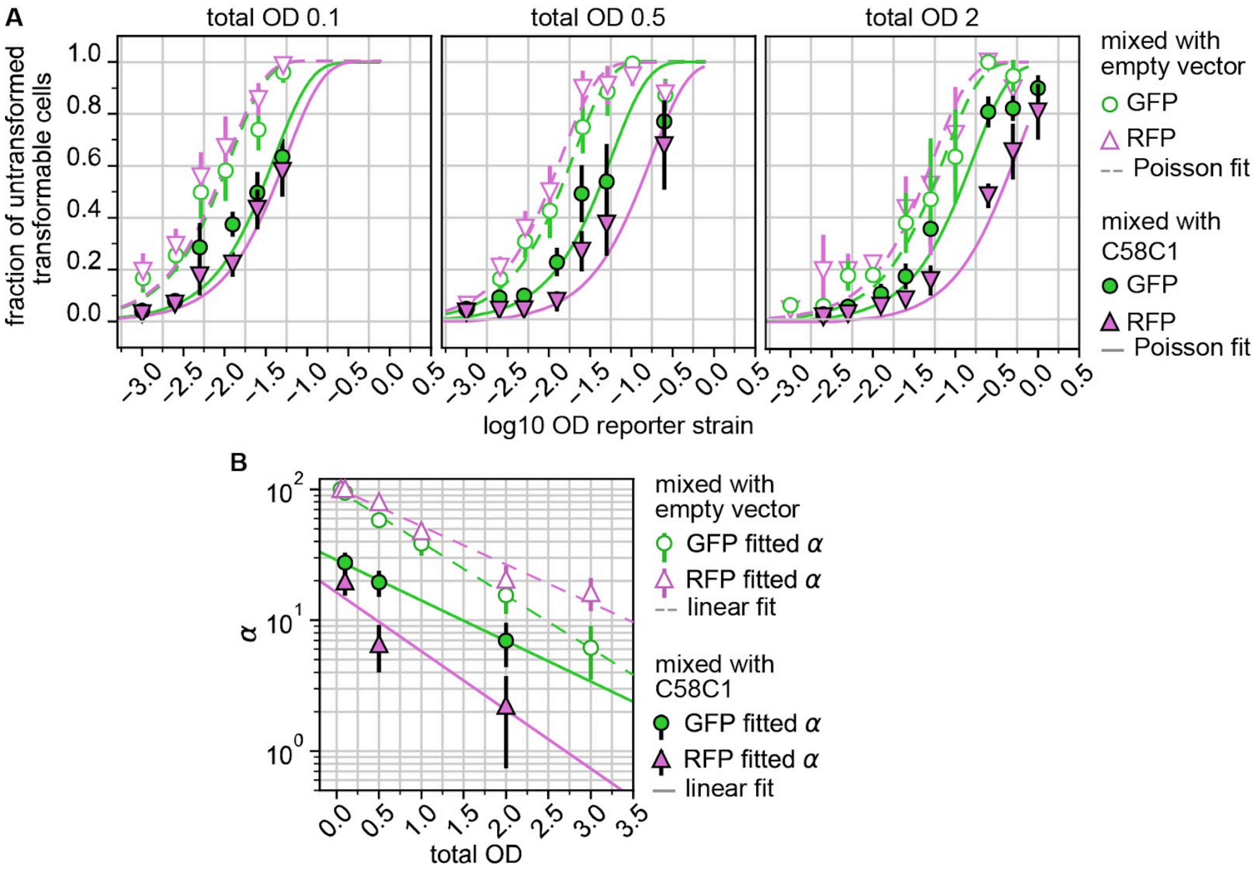

Supplementary Figure 3. Related to Figure 3B: Comparison of transformation rates by reporter strains using EV or C58C1 as the antagonistic strain. (A) The reporter strains were titrated in equal ratios using a third strain to keep the total OD constant, as described in Figure 1C. The third competitor strain used was either EV or C58C1. Shown is the mean ± SEM of the fraction of transformable cells expressing each reporter when EV (empty markers) or C58C1 (filled markers) was used. Dashed and solid lines correspond to Poisson fits to the data for EV and C58C1, respectively. **(B)** The values of α estimated from the fits in (A) are shown as a function of the total OD, as in Figure 2C and 3E. The EV data in (A) and (B) are the same as in Figure 2. In (A) and (B) N = 5 plants (one image per plant) for the C58C1 data.

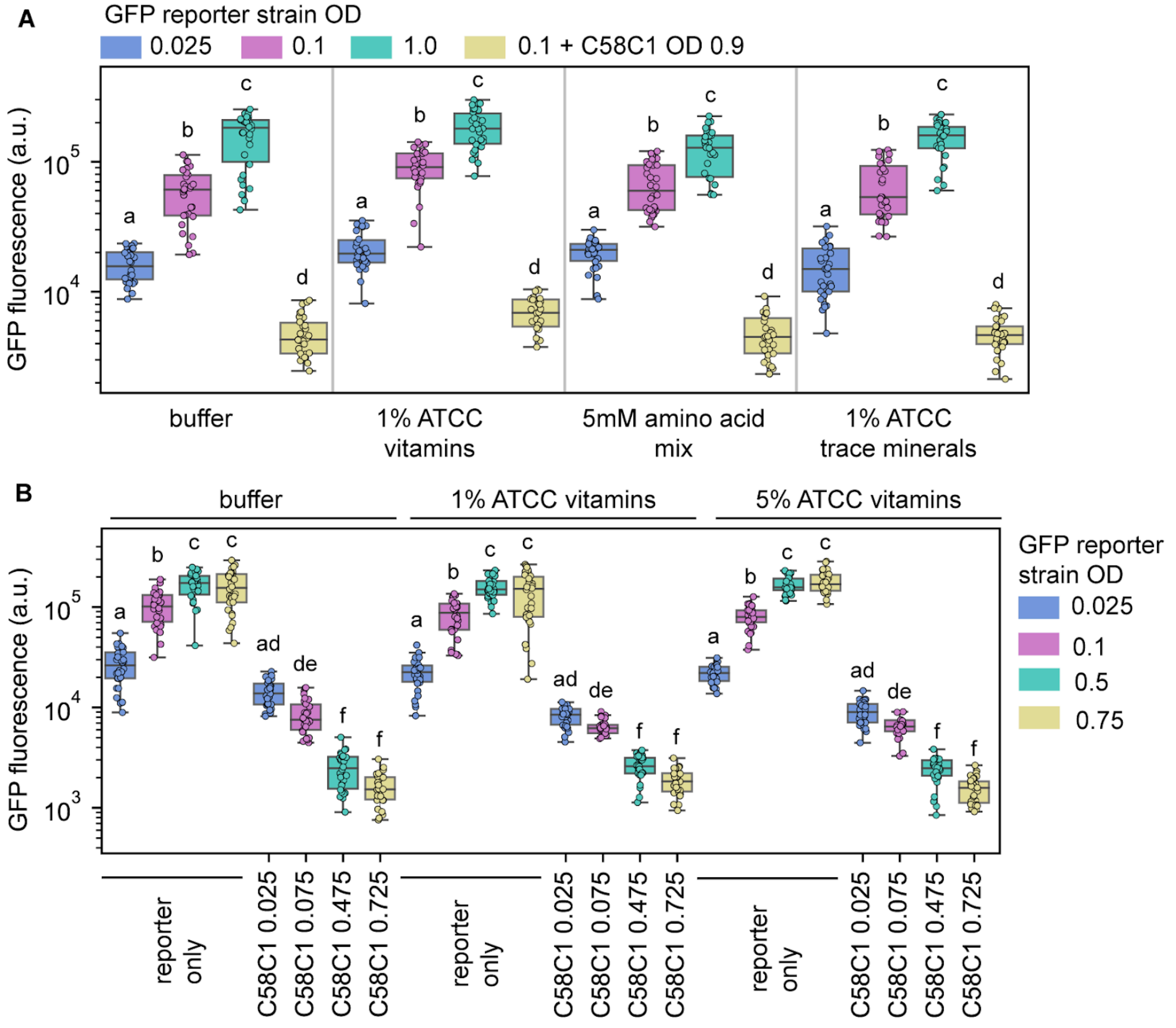

Supplementary Figure 4. Related to Figure 3: Saturation and antagonism cannot be relieved by supplementing the infiltration buffer with vitamins, amino acids or trace minerals. (A) A GFP reporter strain carrying a pCM2-GFP transgene was infiltrated alone at ODs 0.025, 0.1 or 1.0 or at OD 0.1 in combination with C58C1 at OD 0.9. The infiltration buffer was supplemented with 1% v/v ATCC vitamin mix, a 5mM mix of chemically diverse amino acids (E, S, W, L, and C) or 1% v/v ATCC trace minerals. GFP expression was measured using a fluorescence plate reader. **(B)** Similar to **(A)** except that various ODs of C58C1 were tested as

well as two different %v/v concentrations of ATCC vitamins. N = 32 (4 plants, 8 leaf punches per plant). Letters indicate statistically significant ($p < 0.05$) groups based on a post-hoc Tukey HSD test.

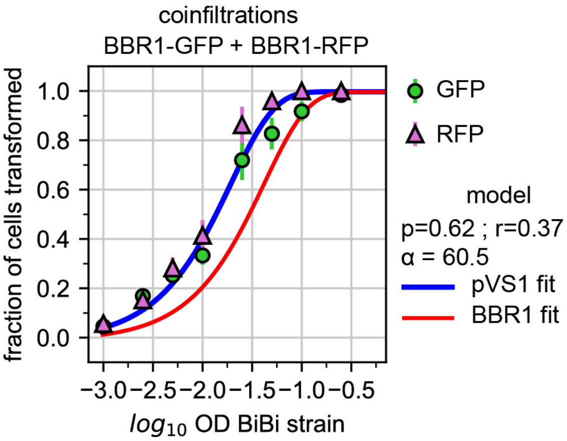

Supplementary Figure 5. Related to Figure 5: The transformation rate of T-DNAs launched from BBR1 is similar to that of pVS1 when BBR1 strains are mixed in the absence of reporter pVS1 strains. Mean ± SD fraction of transformable nuclei expressing the GFP or RFP fluorescence reporters as a function of the infiltration OD of the reporter strain. Both reporter strains carry the BBR1 version of the reporter construct. The total OD was kept constant at 0.5 using EV cells.

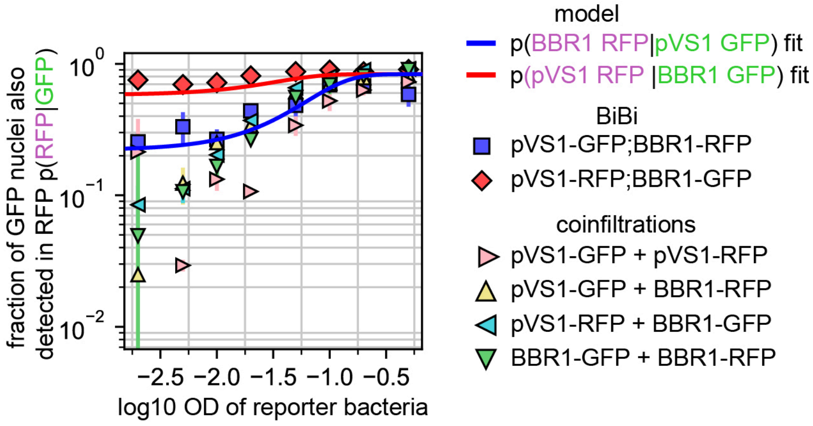

Supplementary Figure 6. Related to Figure 5: Fraction of cells that express RFP given that they also express GFP and fit of the BiBi model to the data. As in figure 5, reporter strains were titrated keeping the total OD constant at 0.5 using the EV strain. Shown is the fraction of nuclei that express GFP that also express RFP (y-axis) as a function of the OD of a BiBi strain or two regular strains combined. The model in Figure 5B was simultaneously fitted to the data shown here as well as data in Figure 5C, D, and E. Shown is the best model fit (solid red and blue lines).

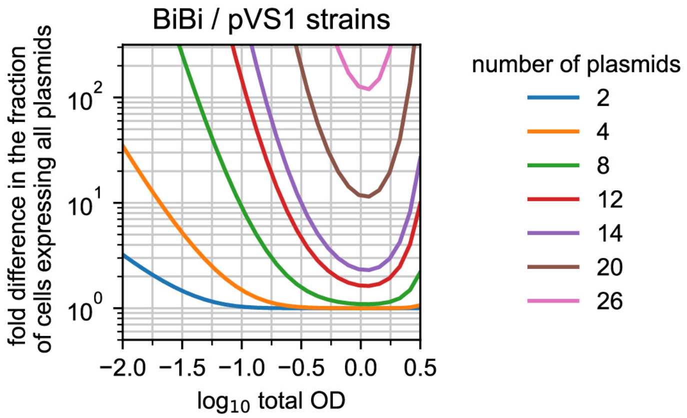

Supplementary Figure 7. Related to Figure 5: predicted fold improvement of BiBi strains versus regular strains in terms of the fraction of plant cells expressing all T-DNAs in a mix. For a mix of N regular pVS1 strains carrying N plasmids we calculated the fraction of plant cells expressing all N T-DNAs when the mix is infiltrated at different total ODs. The same exercise was performed for a mix of N/2 BiBi strains carrying 2 of the N plasmids per strain. Finally, we calculated the ratio in the fraction of plant cells expressing all N T-DNAs between the BiBi prediction and the regular strains prediction (y-axis). The predicted benefit of using BiBi strains gets exacerbated exponentially with an increase in the number of plasmids.

Supplementary Tables

Pathway ID	Gene Name	Organism	Locus
1	BCAT4, BRANCHED-CHAIN AMINOTRANSFERASE4	*A. thaliana*	AT3G19710
2	BAT5, BILE ACID TRANSPORTER 5	*A. thaliana*	AT4G12030
3	MAM1, METHYLTHIOALKYLMALATE SYNTHASE 1	A. thaliana	AT5G23010
4	IIL1, ISOPROPYL MALATE ISOMERASE LARGE SUBUNIT	A. thaliana	AT4G13430
5	IPMI1, ISOPROPYLMALATE ISOMERASE SMALL SUBUNIT	A. thaliana	AT3G58990
6	IMD3, ISOPROPYLMALATE DEHYDROGENASE 1	A. thaliana	AT1G31180
7	BUS1, CYTOCHROME P450 79F1	A. thaliana	AT1G16410
8	REF2, CYTOCHROME P450 83A1	A. thaliana	AT4G13770
9	GSTF11, GLUTATHIONE S-TRANSFERASE F11	A. thaliana	AT3G03190
10	GGP1, gamma-glutamyl peptidase 1	B. rapus	LOC103852592
11	SUR1, S-alkyl-thiohydroximate lyase	B. rapus	LOC106440999
12	AtUGT74C1, UDP-Glycosyltransferase 74C1	A. thaliana	AT2G31790
13	SOT17, SULFOTRANSFERASE 17	A. thaliana	AT1G18590
14	FMO GS-OX, FLAVIN-MONOOXYGENASE GLUCOSINOLATE S-OXYGENASE 1	A. thaliana	AT1G65860

Table S1. Genes used to reconstitute glucoraphanin biosynthesis in *N. benthamiana* in Figure 6 and Supplementary Figure 11.

Plasmid	Origin/Resistance	Description	Reference
PCM2-GFP	pVS1/Kan	Binary vector expressing eGFP from a medium strength constitutive promoter	10.1021/acssynbio.3c00075
PCH5-GFP	pVS1/Kan	Binary vector expressing eGFP from a high strength constitutive promoter	10.1021/acssynbio.3c00075
PCL2-GFP	pVS1/Kan	Binary vector expressing eGFP from a low strength constitutive promoter	10.1021/acssynbio.3c00075
pMQ30K-ΔcelA-celC	pUC/Kan	Used for deletion of genes celA-celC (Atu3307-Atu3309) in C58C1	This work
pMQ30K-ΔUPP	pUC/Kan	Used for deletion of genes Atu1235-Atu1236 in C58C1 and GV3101	This work
pMQ30K-ΔtraI	pUC/Kan	Used for deletion of gene traI in GV3101	This work
UtB2N7	pVS1/Kan	binary vector containing pAtUBQ10::BFP-NLS construct used to transform tobacco	10.1038/s41477-021-00976-0

pCambia1300	pVS1/Kan	empty binary vector	
SAPS514	pVS1/Kan	binary vector containing pAtUBQ10::RFP-NLS used in reporter strains	This work
SAPS614	pVS1/Kan	binary vector containing pAtUBQ10::GFP-NLS used in reporter strains	This work
SAPS654	BBR1/Spec	binary vector containing pAtUBQ10::GFP-NLS used in reporter strains	This work
SAPS656	BBR1/Spec	binary vector containing pAtUBQ10::RFP-NLS used in reporter strains	This work
pGingerBS-LacI-virE12	BBR1/Spec	IPTG inducible complementation plasmid of virE12	(29)
BBR1_At3g03190	BBR1/Spec	Plant Expression Vector For High Level Expression of At3g03190	This work
BBR1_At4g12030	BBR1/Spec	Plant Expression Vector For High Level Expression of At4g12030	This work
BBR1_AtIPMDH	BBR1/Spec	Plant Expression Vector For High Level Expression of AtIPMDH	This work
BBR1_AtIPMI_S	BBR1/Spec	Plant Expression Vector For High Level Expression of AtIPMI_S	This work
BBR1_AtUGT74C1	BBR1/Spec	Plant Expression Vector For High Level Expression of AtUGT74C1	This work
BBR1_BCAT1	BBR1/Spec	Plant Expression Vector For High Level Expression of BCAT1	This work
BBR1_Cyp79F1	BBR1/Spec	Plant Expression Vector For High Level Expression of Cyp79F1	This work
BBR1_Cyp83A1	BBR1/Spec	Plant Expression Vector For High Level Expression of Cyp83A1	This work
BBR1_GGP1_T2A	BBR1/Spec	Plant Expression Vector For High Level Expression of GGP1_T2A	This work
BBR1_GGP1	BBR1/Spec	Plant Expression Vector For High Level Expression of GGP1	This work
BBR1_L_IPMI	BBR1/Spec	Plant Expression Vector For High Level Expression of L_IPMI	This work
BBR1_GS_OX1	BBR1/Spec	Plant Expression Vector For High Level Expression of GS_OX1	This work
BBR1_SUR1	BBR1/Spec	Plant Expression Vector For High Level Expression of SUR1	This work
BBR1_SOT17	BBR1/Spec	Plant Expression Vector For High Level Expression of SOT17	This work
BBR1_SOT17_T2A	BBR1/Spec	Plant Expression Vector For High Level Expression of SOT17_T2A	This work
BBR1_MAM1	BBR1/Spec	Plant Expression Vector For High Level Expression of MAM1	This work
pCH5_At4g1230_pVS	pVS1/Kan	Plant Expression Vector For High Level Expression	This work

1_kan		of At4g12030	
pCH5_At3g03190_pVS1_kan	pVS1/Kan	Plant Expression Vector For High Level Expression of At3g03190	This work
PaqCI_pink_do_pVS1_kan	pVS1/Kan	Used as plasmid intermediate for construction	This work
PaqCI_pink_do_pBBR1_spec	pVS1/Kan	Used as plasmid intermediate for construction	This work
IPMI_L_pVS1_kan	pVS1/Kan	Plant Expression Vector For High Level Expression of L_IPMI	This work
pCH5_Cyp79F1_pVS1_kan	pVS1/Kan	Plant Expression Vector For High Level Expression of Cyp79F1	This work
pCH5_BrSUR1_pVS1_kan	pVS1/Kan	Plant Expression Vector For High Level Expression of SUR1	This work
pCH5_BCAT4_pVS1_kan	pVS1/Kan	Plant Expression Vector For High Level Expression of BCAT1	This work
pCH5_AtCyp83A1_pVS1_kan	pVS1/Kan	Plant Expression Vector For High Level Expression of Cyp83A1	This work
pCH5_At4gUGT74C1_pVS1_kan	pVS1/Kan	Plant Expression Vector For High Level Expression of AtUGT74C1	This work
pCH5_IPMDH_pVS1_kan	pVS1/Kan	Plant Expression Vector For High Level Expression of AtIPMDH	This work
pCH5_GS_OX1_pVS1_kan	pVS1/Kan	Plant Expression Vector For High Level Expression of GS_OX1	This work
pCH5_GGP1_T2A_pVS1_kan	pVS1/Kan	Plant Expression Vector For High Level Expression of GGP1_T2A	This work
pCH5_GGP1_pVS1_kan	pVS1/Kan	Plant Expression Vector For High Level Expression of GGP1	This work
pCH5_SOT17_pVS1_kan	pVS1/Kan	Plant Expression Vector For High Level Expression of SOT17	This work
pCH5_MAM1_pVS1_kan	pVS1/Kan	Plant Expression Vector For High Level Expression of MAM1	This work
pCH5_IPMI_S_pVS1_kan	pVS1/Kan	Plant Expression Vector For High Level Expression of AtIPMI_S	This work
pCH5_IPMI_L_pVS1_kan	pVS1/Kan	Plant Expression Vector For High Level Expression of L_IPMI	This work

Table S2. List of plasmids used in this study

**Strain**	**Description**	**Reference/ Source**
E. coli XL1-Blue	Cloning strain of E. coli	Agilent
E. coli S17	Strain of E. coli used for conjugation	ATCC 47055
Agrobacterium fabrum C58C1	A. fabrum C58 that has been cured of pTiC58	10.1038/252169a0
Agrobacterium fabrum GV3101:pMP90	Laboratory strain of A. fabrum C58 that has been	Intact Genomics

	disarmed of T-DNA	
Agrobacterium fabrum EHA105	Laboratory strain of A. fabrum C58 carrying hypervirulent helper plasmid	Intact Genomics
Agrobacterium fabrum C58C1 ΔcelA-celC	Internal in frame deletion of genes celA-celC in C58C1	This work
Agrobacterium fabrum C58C1 ΔUPP	Internal in frame deletion of genes Atu1235-Atu1236 in C58C1	This work
Agrobacterium fabrum GV3101 ΔUPP	Internal in frame deletion of genes Atu1235-Atu1236 in GV3101	This work
Agrobacterium fabrum GV3101 ΔtraI	Internal in frame deletion of gene traI in GV3101	This work
Agrobacterium fabrum GV3101 ΔvirB1-virB11	Internal in frame deletion of genes virB1-virB11 in GV3101	(29)
Agrobacterium fabrum GV3101 ΔvirD1-virD2	Internal in frame deletion of genes virD1-virD2 in GV3101	(29)
Agrobacterium fabrum GV3101 ΔvirE1-virE2	Internal in frame deletion of genes virE1-virE2 in GV3101	(29)
Pseudomonas syringae pv. *syringae* B728a	Plant pathogenic bacterium	(40)

Table S3. List of Agrobacterium strains used in this study
