# Supplementary material for: Quantitative dissection of Agrobacterium T-DNA expression in single plant cells reveals density-dependent synergy and antagonism
Source: Nat Plants. 2025 May 12;11(5):1060–73. doi: 10.1038/s41477-025-01996-w (PMC12095076; doi:10.1038/s41477-025-01996-w)
Supplement: Supplementary file 2 — Reporting Summary [file 41477_2025_1996_MOESM2_ESM.pdf]

Corresponding author(s): Patrick M. Shih
Last updated by author(s): 2025-3-24

Reporting Summary

Nature Portfolio wishes to improve the reproducibility of the work that we publish. This form provides structure for consistency and transparency in reporting. For further information on Nature Portfolio policies, see our Editorial Policies and the Editorial Policy Checklist.

Statistics

For all statistical analyses, confirm that the following items are present in the figure legend, table legend, main text, or Methods section.

n/a	Confirmed	
☐	☒	The exact sample size (n) for each experimental group/condition, given as a discrete number and unit of measurement
☐	☒	A statement on whether measurements were taken from distinct samples or whether the same sample was measured repeatedly
☐	☒	The statistical test(s) used AND whether they are one- or two-sided<br>*Only common tests should be described solely by name; describe more complex techniques in the Methods section.*
☒	☐	A description of all covariates tested
☐	☒	A description of any assumptions or corrections, such as tests of normality and adjustment for multiple comparisons
☐	☒	A full description of the statistical parameters including central tendency (e.g. means) or other basic estimates (e.g. regression coefficient) AND variation (e.g. standard deviation) or associated estimates of uncertainty (e.g. confidence intervals)
☐	☒	For null hypothesis testing, the test statistic (e.g. F, t, r) with confidence intervals, effect sizes, degrees of freedom and P value noted<br>*Give P values as exact values whenever suitable.*
☒	☐	For Bayesian analysis, information on the choice of priors and Markov chain Monte Carlo settings
☒	☐	For hierarchical and complex designs, identification of the appropriate level for tests and full reporting of outcomes
☐	☒	Estimates of effect sizes (e.g. Cohen's d, Pearson's r), indicating how they were calculated

Our web collection on statistics for biologists contains articles on many of the points above.

Software and code

Policy information about availability of computer code

Data collection	No custom code was used to collect raw data
Data analysis	No new software or custom algorithms were used. Python code used to analyze the data can be found in https://github.com/shih-lab/agro_poisson

For manuscripts utilizing custom algorithms or software that are central to the research but not yet described in published literature, software must be made available to editors and reviewers. We strongly encourage code deposition in a community repository (e.g. GitHub). See the Nature Portfolio guidelines for submitting code & software for further information.

Data

Policy information about availability of data

All manuscripts must include a data availability statement. This statement should provide the following information, where applicable:

- Accession codes, unique identifiers, or web links for publicly available datasets
- A description of any restrictions on data availability
- For clinical datasets or third party data, please ensure that the statement adheres to our policy

Nuclei counts from microscopy experiments, leaf punch fluorescence intensity from plate reader experiments and LC-MS data are available as supplementary tables. All microscopy raw data is available from the authors upon request.

Research involving human participants, their data, or biological material

Policy information about studies with human participants or human data. See also policy information about sex, gender (identity/presentation), and sexual orientation and race, ethnicity and racism.

Reporting on sex and gender	Not applicable.
Reporting on race, ethnicity, or other socially relevant groupings	Not applicable.
Population characteristics	Not applicable.
Recruitment	Not applicable.
Ethics oversight	Not applicable.

Note that full information on the approval of the study protocol must also be provided in the manuscript.

Field-specific reporting

Please select the one below that is the best fit for your research. If you are not sure, read the appropriate sections before making your selection.

☒ Life sciences ☐ Behavioural & social sciences ☐ Ecological, evolutionary & environmental sciences

For a reference copy of the document with all sections, see nature.com/documents/nr-reporting-summary-flat.pdf

Life sciences study design

All studies must disclose on these points even when the disclosure is negative.

Sample size	No statistical methods were used to predetermine sample sizes. In fluorescence plate reader assays, sample sizes were chosen based on known variation between leaves and plants in N. benthamiana and for statistical rigor to detect potential differences between samples, and this size exceeds the standards set by other leading studies in the field that use N. benthamiana as a screening tool (PMID: 35951698, 37348464, 37558215,39496930). In imaging experiments, each data point corresponds to a field of view obtained from one leaf of one plant. In most imaging experiments N was at least 4 plants and more plants were added in cases where the data had a very high dispersion compared to other treatments. In figure 4B and 4C, to better detect potentially subtle effects we used N=9 plants (one image per plant).
Data exclusions	No data was excluded
Replication	Many of our imaging experiments have an internal replication control in the form of two fluorescent proteins (GFP and RFP). We found these two reporters to behave very consistently. Many of our observations are replicated within the manuscript. For example, the positive an negative interactions shown in Fig. 3F can also be seen in Fig. 4A and 4E.. The LC-MS experiment shown in Fig. 6C was performed with fewer replicates and the results were qualitatively consistent (Fig. S11). We conducted the immunity priming assay with P. syringae twice using two different ODs of P. syringae and found qualitatively similar results although only OD 0.004 is shown in the paper. The ROS luminescence assays were also replicable, conducted multiple times on different days using different batches of plants and bacteria.
Randomization	Plants were randomly selected for each treatment across experiments. In imaging experiments, when multiple mixes are infiltrated into the same leaf, the position of the mixes within the leaf was randomized.
Blinding	Because all microscopy samples are imaged simultaneously and imaging settings are the same, no blinding was used during imaging. Images were blinded for downstream computational processing. No blinding was used for plate reader experiments given the nature of these assays: Leaf punches are obtained simultaneously from all treatments and loaded on shared microplates which are then measured using a common standard setup in a plate reader. This applies to the ROS luminescence assays and the LC-MS experiments as well.

Reporting for specific materials, systems and methods

We require information from authors about some types of materials, experimental systems and methods used in many studies. Here, indicate whether each material, system or method listed is relevant to your study. If you are not sure if a list item applies to your research, read the appropriate section before selecting a response.

Materials & experimental systems

n/a	Involved in the study
☒	☐ Antibodies
☒	☐ Eukaryotic cell lines
☒	☐ Palaeontology and archaeology
☒	☐ Animals and other organisms
☒	☐ Clinical data
☒	☐ Dual use research of concern
☐	☒ Plants

Methods

n/a	Involved in the study
☒	☐ ChIP-seq
☒	☐ Flow cytometry
☒	☐ MRI-based neuroimaging

Dual use research of concern

Policy information about dual use research of concern

Hazards

Could the accidental, deliberate or reckless misuse of agents or technologies generated in the work, or the application of information presented in the manuscript, pose a threat to:

No	Yes
☒	☐ Public health
☒	☐ National security
☒	☐ Crops and/or livestock
☒	☐ Ecosystems
☒	☐ Any other significant area

Experiments of concern

Does the work involve any of these experiments of concern:

No	Yes
☒	☐ Demonstrate how to render a vaccine ineffective
☒	☐ Confer resistance to therapeutically useful antibiotics or antiviral agents
☒	☐ Enhance the virulence of a pathogen or render a nonpathogen virulent
☒	☐ Increase transmissibility of a pathogen
☒	☐ Alter the host range of a pathogen
☒	☐ Enable evasion of diagnostic/detection modalities
☒	☐ Enable the weaponization of a biological agent or toxin
☒	☐ Any other potentially harmful combination of experiments and agents

Plants

Seed stocks	Nicotiana benthamiana wild type seeds were obtained from the JBEI feedstocks collection.
Novel plant genotypes	A transgenic Nicotiana benthamiana line carrying a constitutively expressed nuclear-localized blue fluorescent protein (BFP) transgene was generated.
Authentication	The transgenic BFP Nicotiana benthamiana line was authenticated in two ways. First, light microscopy was used to confirm strong expression of the nuclear BFP transgene. Second, the health of the plants was determined by growing them side by side with wild-type N. benthamiana. No obvious phenotypic defects were observed.
